# Identification of Selective Novel Hits against *Plasmodium falciparum* Prolyl tRNA Synthetase Active Site and a Predicted Allosteric Site Using In Silico Approaches

**DOI:** 10.3390/ijms21113803

**Published:** 2020-05-27

**Authors:** Dorothy Wavinya Nyamai, Özlem Tastan Bishop

**Affiliations:** Research Unit in Bioinformatics (RUBi), Department of Biochemistry and Microbiology, Rhodes University, Grahamstown 6140, South Africa; dornyam@gmail.com

**Keywords:** aminoacyl tRNA synthetase, free energy landscape, MD-TASK, dynamic residue network, allosteric modulators, virtual screening

## Abstract

Recently, there has been increased interest in aminoacyl tRNA synthetases (aaRSs) as potential malarial drug targets. These enzymes play a key role in protein translation by the addition of amino acids to their cognate tRNA. The aaRSs are present in all *Plasmodium* life cycle stages, and thus present an attractive malarial drug target. Prolyl tRNA synthetase is a class II aaRS that functions in charging tRNA with proline. Various inhibitors against *Plasmodium falciparum* ProRS (PfProRS) active site have been designed. However, none have gone through clinical trials as they have been found to be highly toxic to human cells. Recently, a possible allosteric site was reported in PfProRS with two possible allosteric modulators: glyburide and TCMDC-124506. In this study, we sought to identify novel selective inhibitors targeting PfProRS active site and possible novel allosteric modulators of this enzyme. To achieve this, virtual screening of South African natural compounds against PfProRS and the human homologue was carried out using AutoDock Vina. The modulation of protein motions by ligand binding was studied by molecular dynamics (MD) using the GROningen MAchine for Chemical Simulations (GROMACS) tool. To further analyse the protein global motions and energetic changes upon ligand binding, principal component analysis (PCA), and free energy landscape (FEL) calculations were performed. Further, to understand the effect of ligand binding on the protein communication, dynamic residue network (DRN) analysis of the MD trajectories was carried out using the MD-TASK tool. A total of ten potential natural hit compounds were identified with strong binding energy scores. Binding of ligands to the protein caused observable global and residue level changes. Dynamic residue network calculations showed increase in betweenness centrality (*BC*) metric of residues at the allosteric site implying these residues are important in protein communication. A loop region at the catalytic domain between residues 300 and 350 and the anticodon binding domain showed significant contributions to both PC1 and PC2. Large motions were observed at a loop in the Z-domain between residues 697 and 710 which was also in agreement with RMSF calculations that showed increase in flexibility of residues in this region. Residues in this loop region are implicated in ATP binding and thus a change in dynamics may affect ATP binding affinity. Free energy landscape (FEL) calculations showed that the holo protein (protein-ADN complex) and PfProRS-SANC184 complexes were stable, as shown by the low energy with very few intermediates and hardly distinguishable low energy barriers. In addition, FEL results agreed with backbone RMSD distribution plots where stable complexes showed a normal RMSD distribution while unstable complexes had multimodal RMSD distribution. The betweenness centrality metric showed a loss of functional importance of key ATP binding site residues upon allosteric ligand binding. The deep basins in average *L* observed at the allosteric region imply that there is high accessibility of residues at this region. To further analyse *BC* and average *L* metrics data, we calculated the Δ*BC* and Δ*L* values by taking each value in the holo protein *BC* or *L* matrix less the corresponding value in the ligand-bound complex *BC* or *L* matrix. Interestingly, in allosteric complexes, residues located in a loop region implicated in ATP binding had negative Δ*L* values while in orthosteric complexes these residues had positive Δ*L* values. An increase in contact frequency between residues Ser263, Thr267, Tyr285, and Leu707 at the allosteric site and residues Thr397, Pro398, Thr402, and Gln395 at the ATP binding TXE loop was observed. In summary, this study identified five potential orthosteric inhibitors and five allosteric modulators against PfProRS. Allosteric modulators changed ATP binding site dynamics, as shown by RMSF, PCA, and DRN calculations. Changes in dynamics of the ATP binding site and increased contact frequency between residues at the proposed allosteric site and the ATP binding site may explain how allosteric modulators distort the ATP binding site and thus might inhibit PfProRS. The scaffolds of the identified hits in the study can be used as a starting point for antimalarial inhibitor development with low human cytotoxicity.

## 1. Introduction

Malaria affects almost one third of the world’s population, mostly in Asia, Africa, and South America [1,2]. Globally, an estimated 219 million malaria cases and approximately 435,000 malaria deaths were reported in 2017 [2]. Parasites of the genus *Plasmodium* are the causative agents for malaria, and the disease is spread by female *Anopheles* mosquitoes. The life cycle of the parasite consists of asexual and sexual blood stages [3,4]. The sexual stage gives rise to gametocytes which transmit the infection from one host to another through *Anopheles* mosquito, while the asexual stage causes the clinical manifestation of malaria [3,5]. Currently, the first-line drugs for malaria treatment comprise five artemisinin-based combination therapies (ACTs) [6]. Artemisinin and its derivatives are sesquiterpene lactones that are active against all *Plasmodium* blood stages [5]. Even though ACTs have yielded recognizable levels of reduction in malaria cases, recently *Plasmodium falciparum* parasite resistance to artemisinin has been reported in South East Asia [1,7]. The future is uncertain as disease surveillance reports indicate the rapid development and spread of resistant *P. falciparum* strains, no matter what drugs are used [8]. Considering the continuous drug resistance of parasites against existing drugs, we need non-conventional approaches to identify drug targets, drug targeting sites (e.g., allosteric hot spots) and new drugs, such as allosteric modulators. To date, there is not one allosteric drug on the market for malaria treatment [9].

Allostery is a change of one site of a protein resulting in a functional change at another distant site through dynamics or conformation. Allosteric drugs provide exciting avenues for development of therapeutic agents as they have many potential advantages over orthosteric drugs [9,10,11]. Allosteric sites are less conserved compared to active sites, therefore allosteric modulators are highly specific, hence may be less toxic to host [10,11,12]. In addition, unlike orthosteric drugs that compete with the substrate and cofactors, allosteric drugs can be active, even in the presence of the native substrates, and thus reduce the chances of parasites developing resistance by increasing substrate concentrations [13]. In cases where the allosteric drugs lack an agonistic effect, and are active only in the presence of the substrate, the spatial and temporal activity of the endogenous substrate is preserved [10,11,13]. Furthermore, studies have shown that allosteric modulators are easily derivatized to improve their activity compared to orthosteric drugs [13].

Recently, enzymes involved in protein synthesis have attracted interest in the design of antiparasitic drugs [1,14,15,16,17,18,19,20]. Aminoacyl tRNA synthetases (aaRSs), which add amino acids to their cognate tRNA during protein translation [14,21], are one of these families of proteins. These enzymes are present in all stages of *Plasmodium* life cycle, and have been validated as drug targets in several microorganisms [20,22,23,24]. Detailed information about aaRSs are given in a number of recent articles [14,21,25,26,27,28]. In this study, our interest is *P. falciparum* prolyl tRNA synthetase (PfProRS) as a potential malarial drug target. We targeted the active site of the enzyme to identify unique and specific hits, and explored a potential allosteric site that was reported in our previous study [13]. To our knowledge, there is only one study to date identifying allosteric modulators against that site [25,29].

*Plasmodium falciparum* has two copies of ProRS enzyme, one that functions in the cytoplasm and another copy that is targeted to the apicoplast [1]. In human, however, this protein is expressed as a bifunctional enzyme (Pro/GluRS) carrying out aminoacylation of both proline and glutamate, and is part of the multi-aminoacyl synthetase complex (MSC) [30,31]. The N-terminal region (residues 1–677) of the bifunctional enzyme is involved in glutamate aminoacylation, while the C-terminal region (residues 1001–1512) functions in aminoacylation of proline [31]. The two catalytic domains are linked by three tandem repeats that are involved in protein-nucleic and protein-protein interactions [31,32]. In both human and *Plasmodium*, ProRS occurs as a dimer with the dimer interface consisting of hydrophobic interactions, salt bridges and polar interactions formed between the catalytic domains [33]. *Plasmodium falciparum* ProRS comprises mainly of four domains: the N-terminal domain (NTD, residues 1–254), catalytic domain (CD, residues 255–532), anticodon binding domain (ABD, residues 533–655), and the C-terminal zinc binding like domain (Z-domain, residues 656–746) [34]. The human homologue (HsProRS) also consists of a 15 residue NTD (residues 1001–1015) which links GluRS and ProRS as this protein occurs as a bifunctional enzyme, a CD (residues 1016–1296), ABD (residues 1297–1423), and the Z-domain (residues 1424–1512) [33].

In PfProRS and HsProRS proteins, the CD is characterized by six antiparallel β-strands flanked by three β-sheets and α-helices. The ABD forms four helices and a β-sheet and is connected to the Z-domain through a long helix region [33]. Alanine and cysteine, just like proline, are uncharged amino acids and their side chains are small enough to fit in the proline binding pocket [35]. To ensure fidelity in protein translation, prokaryotes, archaea, and eukaryotes have a ProRS enzyme with an editing region at the CD that hydrolyzes mischarged proline-tRNA with cysteine (Cys-tRNA^Pro^) and proline-tRNA with alanine (Ala-tRNA^Pro^) [14,26,34,36]. In HsProRS, the Z-domain has a Zn^2+^ ion coordinated by residues Cys1448, Cys1453, Cys1495 and Cys1497 which form part of a zinc binding motif and interacts tightly with the CD [33]. The zinc binding motif is present in HsProRS C-terminus of Z-domain while PfProRS lacks this motif probably due to the substitution of two of the four Zn^2+^ chelating cysteine residues in the human homologue with threonine and serine (Thr686 and Ser732) [33,34]. PfProRS has a high similarity to the human homologue with approximately 66% sequence identity and with some structural deviations at the ABD and Z-domain [29,37]. Notably, ATP binding loop (residues 389–405) with TXE motif shows residue differences between PfProRS and HsProRS and its displacement leads to an open conformation in PfProRS but not in the human homologue [25,34].

Febrifugine, a quinazolinone alkaloid from *Dichroa febrifuga*, was used for treatment of malaria in China for several years but its use as an antimalarial agent was stopped in the mid-1960s due to severe gastrointestinal toxicity [34,37]. Derivatives of the alkaloid were, thus, developed in an attempt to reduce febrifugine cytotoxicity. Halofuginone is a 7-bromo-6-chloro synthetic derivative of febrifugine, and is less toxic to human cells compared to febrifugine [1,38]. Halofuginone is currently used for the treatment of *Cryptosporidium* infections and *Eimeria*, a coccidian parasite [37,39]. Recent studies have shown halofuginone as a potential inhibitor of fibrotic disease, cancer and scleroderma [40,41,42,43]. The molecular target for halofuginone was recently identified as ProRS where it competes with proline for the active site [33,38,44]. The piperidine ring of halofuginone occupies the same site as the pyrrolidine ring of proline while the quinazolinone group binds to the A76 adenosine pocket, thus halofuginone binds to both the proline and tRNA binding pockets of ProRS [44]. Binding of halofuginone is promoted by ATP although binding takes place even in the absence of ATP [33,38]. Residues involved in binding of halofuginone are 100% conserved between PfProRS and HsProRS explaining the cytotoxicity effects observed in human cells [29]. Hewitt et al. reported a probable allosteric pocket adjacent to ATP binding site that is formed by residues 261–272, 276–287, and 513–524 [29]. Glyburide and TCMDC-124506 inhibitors have been reported to selectively inhibit PfProRS through binding at this proposed allosteric site [29]. The inhibitory activity (IC_50_) of glyburide and TCMDC-124506 allosteric modulators against PfProRS has been reported to be 34 μM and 74 μM, respectively, while the inhibitory activity against HsProRS was reported to be less than 40% up to the limit of solubility [29]. This site was also identified as a potential ligand binding site using cavity identification methods in an earlier study by Nyamai and Tastan Bishop [25].

This article presents a further study on ProRS as a malarial drug target with an aim of identifying novel selective compounds against either PfProRS active site or the predicted allosteric site. Virtual screening of 623 South African Natural Compound Database (SANCDB) [45] compounds was carried out via molecular docking against the homology models of PfProRS and HsProRS. A total of five orthosteric inhibitors and five allosteric modulators selective against PfProRS were identified. The effect of ligand binding on the protein stability and communication was studied through molecular dynamics (MD) simulations, principal component analysis (PCA), free energy landscape (FEL) calculations and dynamic residue network analysis (DRN) calculations. All selected hit compounds were stable after 200 ns MD simulations. Allosteric modulators caused significant increase in communication between the allosteric site residues and residues at the active site as shown by DRN and weighted contact map calculations. Overall, this study reports ten natural compounds as potential novel inhibitors for PfProRS that can be used as scaffolds for the further development of antimalarial drugs.

## 2. Results and Discussion

In this study, 632 compounds from SANCDB (https://sancdb.rubi.ru.ac.za/) were docked against PfProRS and HsProRS proteins with the aim of identifying potential hit compounds that either selectively bind to the active site of the parasite protein or to the previously identified potential allosteric site. Ten compounds were identified with these criteria. Prior to docking, the docking parameters were validated by redocking TCMDC-124506 ligand to the protein (PBD ID: 4WI1). Similar interactions were observed for the co-crystallized ligand as with the redocked ligand, with an RMSD value of 0.47 Å between the co-crystallized ligand and the best redocked pose (Supplementary Figure S1). Redocking is considered successful when the RMSD value of ≤2.0 Å is obtained [46]. Ligand-protein interaction analysis and RMSD calculation results, thus, indicated that the parameters used in this study were suitable for molecular docking studies. Protein complexes with the identified hit compounds were, then, further analyzed to see the changes in the protein structure at global and residue levels. Where applicable, the results were compared with the known inhibitors, namely halofuginone—an orthosteric compound, as well as glyburide and TCMDC-124506—allosteric inhibitors.

### 2.1. Five SANCDB Compounds Are Identified for PfProRS Active Site

**Figure 1:**
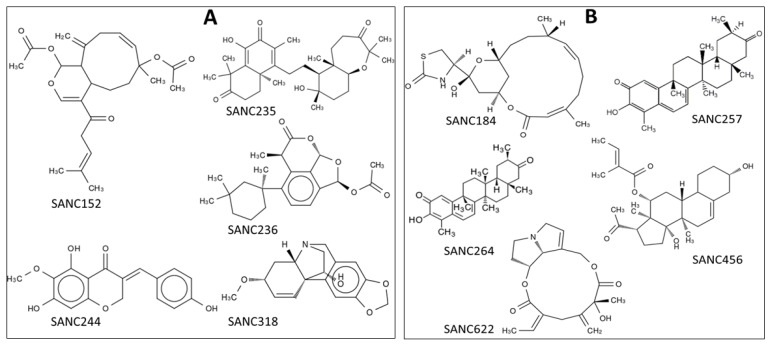
2D representations of identified South African natural compound hits against PfProRS active and allosteric sites. (**A**) Identified orthosteric hit compounds against PfProRS. (**B**) Identified allosteric hit compounds against PfProRS allosteric site.

**Figure 2:**
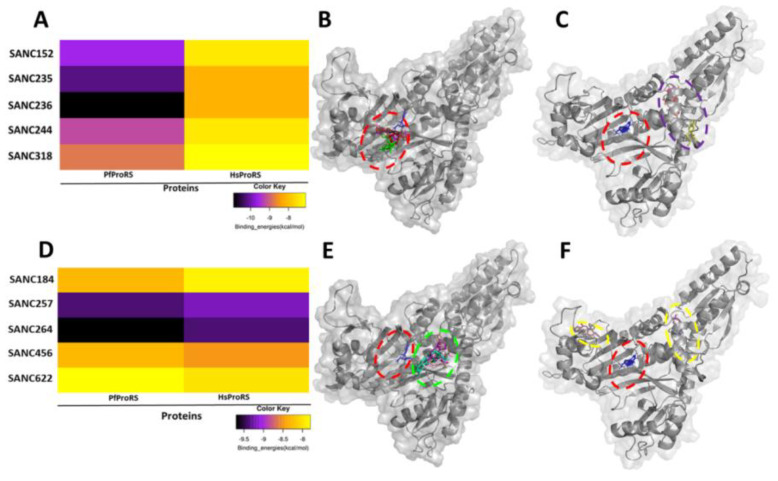
Binding energy (kcal/mol) heat maps and binding modes of the selected orthosteric and allosteric hit compounds on PfProRS and HsProRS. (**A**) Binding energies of orthosteric hit compounds. Selected orthosteric hits binding at the PfProRS active site are shown at the left side of the heat map. Binding energy increases from black to yellow. A black colour shows good binding energy. (**B**) Binding modes of identified orthosteric hit compounds on PfProRS. (**C**) The binding modes of the selected orthosteric hits in HsProRS. In HsProRS, selected orthosteric ligands do not bind the targeted active and allosteric sites. (**D**) Binding energies of allosteric hit compounds. Selected allosteric hits binding at the PfProRS allosteric site are shown at the left side of the heatmap. A black colour shows good binding energy. (**E**) Binding modes of identified allosteric hit compounds on PfProRS. (**F**) Binding poses of identified allosteric hits in HsProRS. Selected allosteric hits do not bind the targeted active and predicted allosteric site in HsProRS. Adenosine binding pose at the active site is shown in blue sticks. The active site is shown in red ellipse while the identified allosteric site is shown in green ellipse. Binding modes of identified orthosteric and allosteric hits on HsProRS are shown in purple and yellow ellipses, respectively.

**Figure 3:**
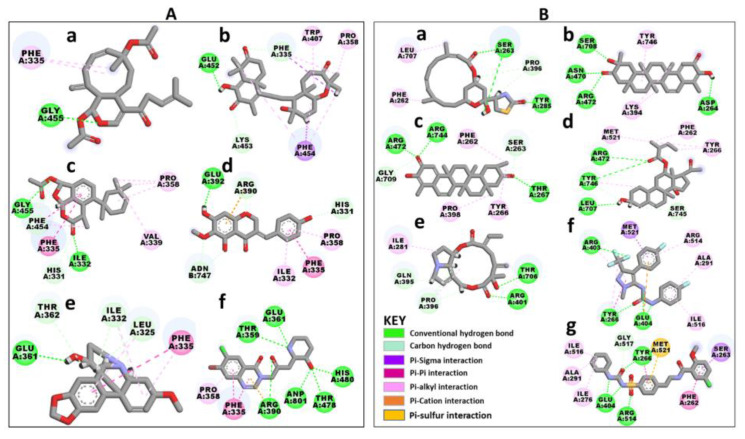
Binding modes of selected (A) orthosteric and (B) allosteric hits. (**A**) 2D representation of (a) SANC152; (b) SANC235; (c) SANC236; (d) SANC244; (e) SANC318; (f) halofuginone (PDB ID: 4YDG) [37] and their binding modes with PfProRS active site. (**B**) 2D representation of (a) SANC184; (b) SANC257; (c) SANC264; (d) SANC456; (e) SANC622 (f) TCMDC-124506 (PDB ID: 4WI1); (g) glyburide (PDB ID: 5IFU) and their binding mode with PfProRS allosteric site.

Five SANCDB compounds, SANC152, SANC235, SANC236, SANC244 and SANC318, were selected (Table 1 and Figure 1) for further analysis as potential orthosteric hits based on their binding energy scores (Table 1 and Figure 2), their selectivity towards the *Plasmodium* protein (Figure 2), and a number of hydrogen bonds that they make with the protein residues (Figure 3). SANC152 is a terpenoid extracted from *Capnella thyrsoidea* [47]. SANC235 and SANC236 are also terpenoids extracted from *Axinella weltneri* and *Aplysilla sulphurea* respectively, and have been reported to have anticancer and antitumor activity on both breast and prostate cancer cell lines [48,49]. SANC244 is a flavonoid from *Eucomis autumnalis* [50]. SANC318, an alkaloid, has been reported to inhibit acetylcholinesterase, and is present in *Crinum bulbispermum* [51].

In the human homolog, these hit compounds did not bind to the active site and the identified potential allosteric site but located at a region formed by the CD and ABD (Figure 2). Active site cavity residues Ile332, Gly334, and Gly455 implicated in binding of the selected orthosteric hits in PfProRS correspond to Val1094, Asp1096, and Ala1217, respectively, in the human homolog (Supplementary Figure S2). These residue differences between PfProRS and HsProRS might explain why these compounds only bound to the PfProRS active site. As the selected orthosteric hit compounds were only binding to PfProRS active site, analysis of interactions and binding poses was only done for PfProRS protein (Figure 3A).

**Figure 4:**
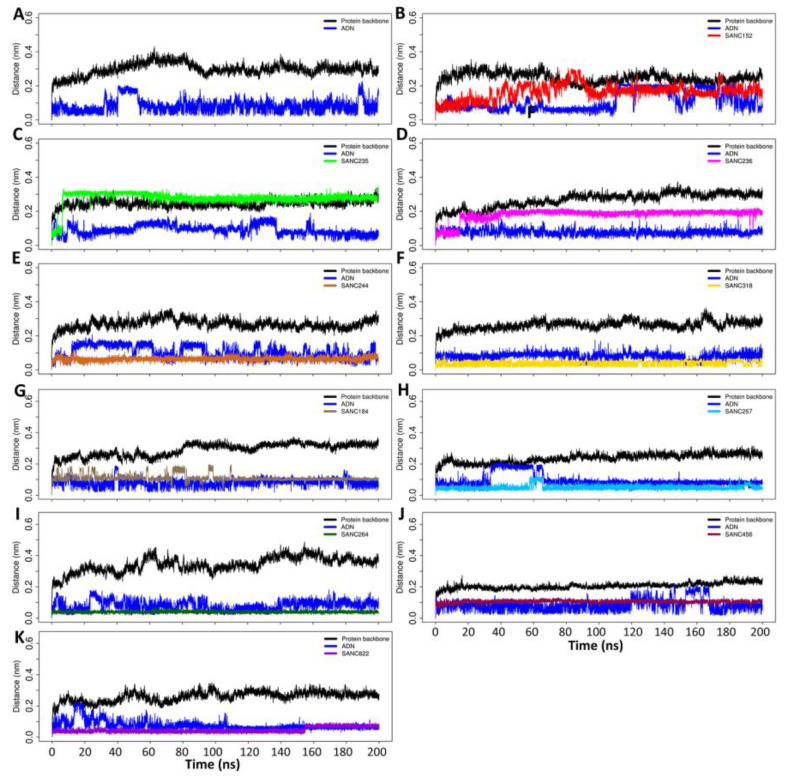
PfProRS backbone RMSD analysis of all atom MD trajectories for the holo system and PfProRS-ligand complexes during the 200 ns simulation. (**A**) Holo system (PfProRS-ADN), (**B**) PfProRS-SANC152 complex, (**C**) PfProRS-SANC235 complex, (**D**) PfProRS-SANC236 complex, (**E**) PfProRS-SANC244 complex, (**F**) PfProRS-SANC318 complex, (**G**) PfProRS-SANC184 complex, (**H**) PfProRS-SANC257 complex, (**I**) PfProRS-SANC264 complex, (**J**) PfProRS-SANC456 complex and (**K**) PfProRS-SANC622 complex.

Further, selected orthosteric hits were compared with halofuginone, showing common interaction patterns with this known orthosteric inhibitor (Figure 3A). Halofuginone, a derivative of febrifugine compound isolated from the roots of *Dichroa febrifuga* is active against *Cryptosporidium* and *Eimeria* infections [39]. The molecular target of halofuginone was first reported in human to be the ProRS enzyme [33,38,44] and its mechanism of action is by inhibition of proline utilization [1,37,38]. In *P. falciparum*, the cellular target of halofuginone is also the ProRS enzyme [34,37]. It binds at the proline binding site and a sub-site occupied by the 3′-end of tRNA [37]. Despite halofuginone activity against PfProRS, the compound has not been approved as antimalarial agent as it has adverse cytotoxic effects on human [34,37]. These adverse effects result from poor selectivity as residues implicated in halofuginone binding are 100% identical in PfProRS and HsProRS [34,37]. In PfProRS, halofuginone inhibitor forms hydrogen bond interactions with Thr359, Glu361, Arg390, Thr478 and His480 (Figure 3A) [37]. Further, the inhibitor forms π-π and π-alkyl interactions with residues Phe335 and Pro358, respectively (Figure 4A) [37]. In this study, all selected ligands showed hydrogen bond interactions and hydrophobic interactions with PfProRS active site residues (Figure 3A). Analysis of binding poses identified a hydrogen bond interaction between SANC152 and residue Gly455 which was also seen in SANC236 interactions. Phe335 formed π-π stacked interactions with ligands SANC152, SANC236, SANC244 and SANC318. This interaction was also observed in halofuginone binding [34,37]. SANC235 formed π-alkyl interactions with Pro358 andTrp407; SANC236 also formed π-alkyl interactions with Val339 and Pro358. SANC244 made a π-cation interaction with Arg390 while the interaction with this residue and halofuginone is a hydrogen bond interaction [34]. SANC244 formed alkyl interactions with residues Ile332 and Pro358 and a carbon-hydrogen bond with His331. SANC318 formed carbon-hydrogen bonds with Leu325, Ile332 and Thr362. A hydrogen bond interaction observed between Glu361 and SANC318 was also reported in halofuginone binding [34,37].

### 2.2. Five Further SANCDB Compounds Are Identified for PfProRS Allosteric Site

In our previous study, we predicted a potential allosteric site in the ProRS enzyme using FTMap and SiteMap [25]. This pocket, located adjacent to the ATP binding site, and formed by residues 261–272, 276–287 and 513–524, was also proposed in a previous study by Hewitt et al. [29]. Virtual screening of SANCDB compounds by molecular docking resulted in five ligands that selectively bind to the PfProRS predicted allosteric site with good binding energy scores and a number of hydrogen bonds (SANC184, SANC257, SANC264, SANC456, and SANC622) (Table 1, Figure 1 and Figure 2). Further, these elected allosteric hits showed similar binding poses as known allosteric inhibitors—TCMDC-124506 and glyburide (Figure 3B).

SANC184 is a diterpene isolated from *Chromodoris hamiltoni* [52] while SANC257 and SANC264 are triterpenes present in *Elaeodendron croceum*, and are reported to have anticancer activity as the compounds are highly cytotoxic [53]. SANC456 is a glycoside that is used as an appetite suppressant, and is present in *Hoodia* species [54]. SANC622 is a pyrrolizidine alkaloid isolated from *Senecio pterophorus* [55].

Although some of these five compounds showed better binding energies in the human homologue, these compounds did not bind to allosteric pocket but again to a site formed by the CD and the ABD as we observed with selective orthosteric compounds (Figure 2). Residues Phe262, Ser263, Thr267, Gln395, Asn470, Thr706, Leu707, and Gly709 implicated in binding of allosteric hits in PfProRS correspond to residues Leu1024, Ala1025, Ser1029, Ser1032, His1157, Ala1472, Pro1473, and Met1474 respectively in HsProRS (Supplementary Figure S2). Selective binding of allosteric hits can be attributed to these residue differences at the proposed allosteric site. As reported in our previous study, this site is characterized by some unique motifs [25]. Although these motifs are present in both PfProRS and HsProRS, differences were observed in residue levels implicated in the binding of FTMap probes and the selected modulators [25].

Analysis of docking poses of allosteric hits showed that the selected ligands had at least two hydrogen bond interactions with PfProRS (Figure 3B). SANC184 had two hydrogen bond interactions with the protein through residues Ser263 and Tyr285. Phe262 and Leu707 made π-alkyl interactions with SANC184 while Pro396 interacted with the ligand through a carbon-hydrogen bond. Asp264, Asn470, Arg472 and Ser708 formed hydrogen bond interactions with SANC257 while Lys394 and Try396 formed π-alkyl interactions with the ligand. Thr267, Arg472, and Arg744 formed hydrogen bond interactions with SANC264 while π-alkyl interactions were observed between residues Phe262, Tyr266, Pro398, and the ligand. SANC456 interacted with Arg472, Leu707, and Tyr746 through hydrogen bonds and to Phe262, Tyr266, and Met521 through π-alkyl interactions. SANC622 had hydrogen bond interactions with residues Arg401 and Thr706, a π-alkyl interaction with Ile281 and carbon-hydrogen interactions with Gln395 and Pro396. Residue Arg401 has also been reported to stabilize the phosphate groups in adenosine and AMPPNP, an ATP analogue [37].

Glyburide and TCMDC-124506 have been reported as allosteric inhibitors that bind selectively to PfProRS at a site adjacent to the ATP binding site [29]. TCMDC-124506 forms three hydrogen bonds with residues Arg403, Glu404, and Tyr266 of the protein by interacting with the urea group [29]. Met521 and Arg514 interacts with TCMDC-124506 head group through a π-sigma and π-π interactions respectively [29]. TCMDC-124506 tail region forms π-alkyl interactions with residues Ala291 and Ile516 [29]. Glyburide also makes hydrogen bonds to Tyr266, Arg514 and Glu404 through the urea group. Phe262 and Ser263 interact with Glyburide’s tail group through π-sigma and π-π stacked interactions respectively. Ala291, Ile276 and Ile516 all form alkyl hydrophobic interactions with glyburide [29]. These residue interactions are also observed in the compounds selected in this study (Figure 3B).

### 2.3. Ligand Binding Modulates Global Protein Motions

To undertand the effect of ligand binding on the global protein motions, following 200 ns MD simulations of the holo protein and protein complexes with identified orthosteric and allosteric hits, the protein backbone RMSD, Rg, PCA, and FEL were calculated.

#### 2.3.1. Root Mean Square Deviations (RMSD)

RThe backbone RMSD provides information on the conformational changes of holo protein and protein-ligand complexes over the simulation period. The ligand RMSD, on the other hand, gives insights on how stable the ligand is over the simulation time. Here, both backbone RMSD and ligand RMSD values were calculated.

**Figure 5:**
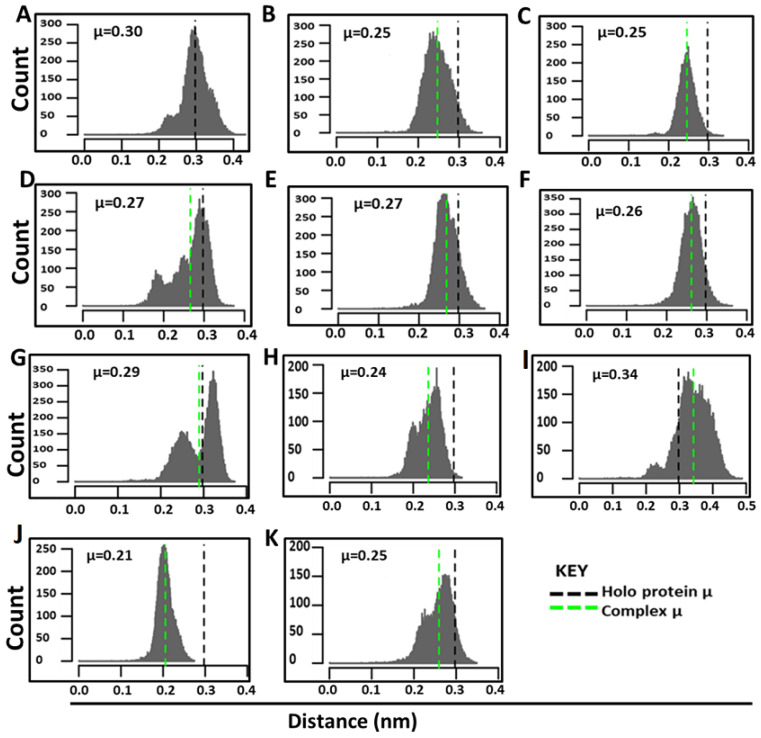
Backbone RMSD distribution plots for PfProRS ligand-bound complexes. Conformational flexibility can be assessed by comparing the backbone RMSD distribution of each ligand-bound complex and the holo protein. Comparison of the mean (µ) of the holo protein (black dashed line) to each ligand complex (green dashed line) demonstrates the shift in conformation distribution of the complexes during the 200 ns MD simulation. σ is the standard deviation. (**A**) Holo system (PfProRS-ADN), (**B**) PfProRS-SANC152 complex, (**C**) PfProRS-SANC235 complex, (**D**) PfProRS-SANC236 complex, (**E**) PfProRS-SANC244 complex, (**F**) PfProRS-SANC318 complex, (**G**) PfProRS-SANC184 complex, (**H**) PfProRS-SANC257 complex, (**I**) PfProRS-SANC264 complex, (**J**) PfProRS-SANC456 complex and (**K**) PfProRS-SANC622 complex.

Root mean square deviation calculation identified a slight shift in backbone conformation in the holo system at 90 ns from 0.39 nm to 0.3 nm during the 200 ns simulation (Figure 4A). In the holo system, adenosine had a RMSD value of 0.05 nm with a structure flip at 40 ns of the simulation (Figure 4A). Root mean square deviation calculations of PfProRS-SANC152 complex showed that the protein backbone slightly changed its conformation from the initial structure by a 0.1 nm deviation (Figure 4B). SANC152 ligand RMSD flipped between 30–80 ns of the simulation (Figure 4B). Adenosine in this complex was stable until 110 ns, after which a flip was observed with conformation changes by 0.15 nm. At 175 ns adenosine went back to the original conformation (Figure 4B). A deviation in conformation of approximately 0.3 nm was seen for both the protein backbone and SANC235 ligand in PfProRS-SANC235 system while adenosine ring flipped at 10 ns, 123 ns, and 155 ns (Figure 4C). RMSD calculations of PfProRS-SANC236 complex showed a 0.2 nm change in backbone conformation, 0.15 nm change for SANC236 ligand while adenosine remained stable through out the simulation period (Figure 4D). PfProRS-SANC244 complex RMSD calculations showed a 0.1 nm deviation in the conformation of the protein backbone while the ligand remained stable with a RMSD of 0.05 nm throughout the simulation (Figure 5E). On the other hand, adenosine structure flipped during the simulation with slight deviation from the initial structure of 0.05 nm (Figure 4E). In PfProRS-SANC318 complex, a slight deviation in the protein backbone conformation of 0.15 nm was observed while SANC318 ligand and adenosine remained stable across the simulation with a RMSD of 0.02 nm and 0.09 nm respectively (Figure 4F).

In the allosteric site, RMSD calculations of PfProRS-SANC184 system showed a deviation in the protein backbone conformation of about 0.25 nm from the initial structure (Figure 4G). In the first 110 ns of the simulation, SANC184 ligand was seen to flip but remained stable for the rest of the simulation with a RMSD of 0.1 nm (Figure 4G). On the other hand, the adenosine structure remained stable throughout the simulation (Figure 4G). The protein backbone in PfProRS-SANC257 complex showed a slight conformational change with a deviation of 0.1 nm at 80 ns while the ligand structure remains stable during the simulation except for a structural flip at 60 ns (Figure 4H). Interestingly, adenosine showed a change in conformation between 30–65 ns with a deviation of approximately 0.1 nm after which it changed to its initial conformation (Figure 4H). RMSD calculations of PfProRS-SANC264 complex indicated a change in conformation of the protein backbone structure from 0.1 nm to 0.3 nm (Figure 4I). SANC264 structure was stable during the 200 ns simulation with a RMSD close to zero while adenosine showed slight deviation from 0 ns to 50 ns then the structure remained stable to the end of the simulation (Figure 4I). The protein backbone and SANC456 ligand in the PfProRS-SANC456 system demonstrated insignificant changes in conformation while adenosine structure showed slight deviation only between 120 and 170 ns (Figure 4J). RMSD calculations of PfProRS-SANC622 showed insignificant deviation of the protein backbone conformation while the ligand structure was stable throughout the simulation period (Figure 4K). Adenosine structure was unstable for the first 70 ns in this system (Figure 4K).

As a control, 100 ns MDs of known inhibitors (halofuginone, glyburide and TCMDC-124506) in complex with PfProRS were carried out. In the three complexes, the backbone RMSDs stabilized at 20 ns of the simulation (Figure S3). This observation was in agreement with the backbone RMSDs of selected hit complexes which were stable throughout the simulation (Figure 4). In all the complexes, adenosine showed a deviation in RMSD at 40 ns from 0.1 nm to 0.2 nm, then the structure flips back to the initial conformation at 55 ns (Supplementary Figure S3A–C). Similarly, adenosine structure flips were observed at different time points of the ligand complexes of our selected hit compounds (Figure 4). In PfProRS-glyburide complex, the ligand showed a structure flip between 40 ns and 65 ns resulting to a decrease in RMSD from 0.4 to 0.3 nm (Supplementary Figure S3A). This structure flip can be attributed to the structure of glyburide which consists of a head group connected to a tail region (Figure 3B). Notably, halofuginone and TCMDC-124506 ligands were stable throughout the simulation (Supplementary Figure S3).

Although the selected orthosteric and allosteric compounds did not bind to the targeted sites in human protein, still the MD simulations were performed to study the possibility of allosteric effects by these compounds on HsProRS. Among the selected orthosteric hits, SANC152, SANC236, and SANC244; and allosteric hits, SANC184 and SANC257 were making stable complex with HsProRS during 200 ns simulations. RMSD calculations of HsProRS holo protein showed that the protein backbone was stable throughout the 200 ns simulation with a RMSD value of 0.2 nm (Figure S4A). In the holo system, adenosine remained stable during the 200 ns simulation with an RMSD value of 0.1 nm. In HsProRS-SANC152 complex, the protein backbone RMSD was stable during the simulation with a RMSD value of 0.2 nm while adenosine remained stable during the 200 ns simulation with a RMSD of 0.1 nm (Supplementary Figure S4B). SANC152 was stable during the simulation with a RMSD value of 0.15 nm (Supplementary Figure S4B). The protein backbone of HsProRS-SANC184 system was stable with a RMSD value of 0.2 nm while adenosine had a RMSD of 0.05 nm (Supplementary Figure S4C). SANC184 underwent structure flips during the 200 ns simulation with a RMSD value ranging between 0.05–0.15 nm (Supplementary Figure S4C). In HsProRS-SANC236 system, at 20 ns there was a shift in protein backbone RMSD from 0.2 nm to 0.3 nm which then remained stable (Supplementary Figure S4D). In this system, adenosine was unstable during the 200 ns with RMSD value ranging from 0.01–0.2 nm (Figure S4D). SANC236 ligand was unstable during the 20 ns simulation with structure flips at 10, 30, 95, 160, and 180 ns of the simulation (Supplementary Figure S4D). In HsProRS-SANC244 system, the protein backbone had a RMSD value of 0.2 nm, adenosine 0.1 nm and SANC244 ligand had a RMSD value of 0.5 nm and were all stable during the 200 ns simulation (Supplementary Figure S4E). The protein backbone in HsProRS-SANC257 system had a RMSD value of 0.15 nm for the first 120 ns then underwent a shift in RMSD to 0.2 nm for the remaining time of the simulation (Supplementary Figure S4F). In this system, adenosine and SANC257 remained stable during the 200 ns simulation with RMSD values of 0.5 nm (Supplementary Figure S4F).

To observe potential discrete conformational changes in proteins with the ligand binding, RMSD distribution histograms of the holo protein and protein complexes, for both orthosteric and allosteric inhibitors, were generated, as previously applied by Penkler and Tastan Bishop [56] (Figure 5). The x-axis, histogram width, presents the number of conformations sampled by proteins, and the y-axis, count, represents number of times a specific conformation was sampled during the MD simulation; hence, the histogram peaks give a representative of the most commonly occupied conformation. All the compounds except allosteric SANC264 compound caused a slight decrease in backbone conformation flexibility as shown by the shift in distribution of the backbone conformation to the left each with mean RMSDs ranging from 0.21–0.29 nm compared to the holo protein which had a mean RMSD of 0.30 nm (Figure 5). Binding of SANC264 appeared to increase conformational flexibility as shown by the mean RMSD of 0.34 nm. PfProRS-SANC236 complex showed a multimodal conformation distribution, while PfProRS-SANC184 system presented a distinct bimodal backbone conformation distribution with a slight shift in mean RMSD (0.29 nm) to the left compared to the holo protein. This distribution implies that binding of SANC184 and SANC236 caused a conformational change of the protein backbone. Mean RMSD values were also compared as average % differences between the holo protein and protein-ligand complexes, with the highest difference observed in PfProRS-SANC456 (30%) for malarial protein (Table 2). The mean RMSD values of PfProRS-glyburide, PfProRS-halofuginone and PfProRS-TCMDC124506 were 0.28, 0.25, and 0.23 nm respectively (Supplementary Figure S5). PfProRS-TCMDC124506 had the highest average % difference of 23.33% for the inhibitor complexes (Table 2).

HsProRS holo protein had a normal backbone conformation distribution with a mean RMSD value of 0.20 nm (Supplementary Figure S6, Table 2). HsProRS-SANC236, HsProRS-SANC244 and HsProRS-SANC257 showed bimodal distributions. The highest average % difference in the mean value of 55% to holo protein was observed in HsProRS-SANC236 complex (Table 2).

#### 2.3.2. Ligand Binding Has No Effect on Protein Backbone Compactness

Binding of the orthosteric and allosteric ligands to PfProRS did not show significant changes in compactness of the protein backbone as shown by plots on radius of gyration (Figure S7). In all the systems, a radius of gyration of between 2.6–2.65 nm was recorded for both the holo protein and the protein ligand complexes (Supplementary Figure S7). Similarly, binding of ligands to HsProRS had no significant effect on protein backbone compactness (Supplementary Figure S8). In all HsProRS ligand complexes, the radius of gyration ranged between 2.58–2.68 nm (Figure S8). Similarly, known PfProRS orthosteric and allosteric inhibitors had no significant effect on the protein backbone compactness as shown by radius of gyration calculations (Supplementary Figure S3D–F). In the inhibitor complexes, the radius of gyration was approximately 2.60 nm (Supplementary Figure S3D–F).

#### 2.3.3. Principal Component Analysis and Free Energy Landscapes

Molecular dynamic simulations generate trajectories that describe protein motions over a broad range of spatial and time scales, thus enabling sampling of conformational ensemble over the total degrees of freedom [57]. Protein function can be studied through interpretation of MD trajectories by extracting structural conformations across the simulation. However, the sampling of structures over the trajectory has to be representative of all the conformations that are accessible to the protein [57]. In addition to RMSD calculations, PCA and free energy landscapes were calculated to further analyze the 3D conformational sampling and internal dynamics of the holo protein and protein–ligand complexes. The percentage variances of the first five principal components for the holo protein and the ligand-bound complexes are summarized in Supplementary Table S2. The protein motions of all atom MD simulation are shown along the first and second principal components (PC1 and PC2) in Supplementary Figure S9.

**Figure 6:**
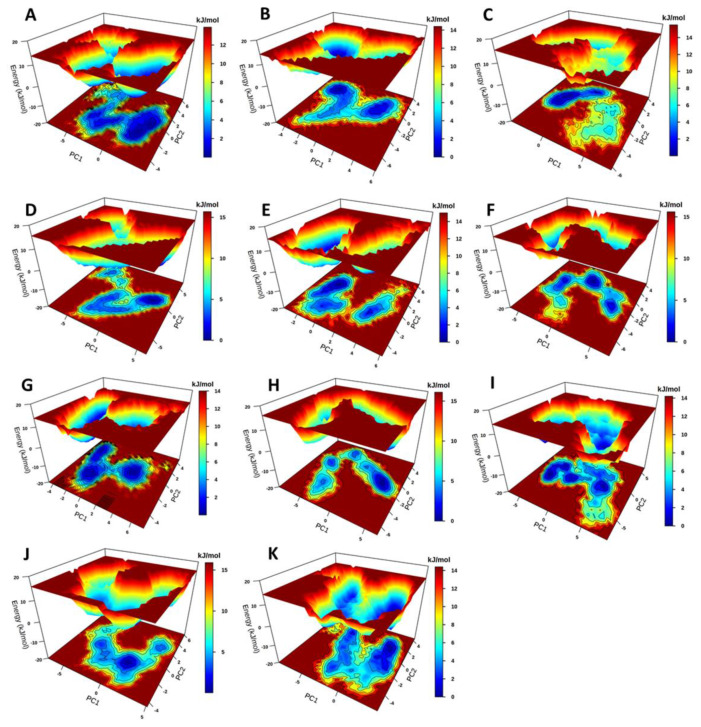
3D representation of binding free energy landscape as a function of PC1 and PC2. Energy distribution is shown by the coloring pattern: Blue defines the conformational space with minimum energy (stable state) while red defines a conformational space with maximum energy (unstable state). Transient local energy states are defined by intermediate color patterns. PC1 and PC2 are displayed as a contour map at the bottom of each FEL plot with similar color pattern like the energy landscape. (**A**) Holo protein (PfProRS-ADN), (**B**) PfProRS-SANC152 complex, (**C**) PfProRS-SANC235 complex, (**D**) PfProRS-SANC236 complex, (**E**) PfProRS-SANC244 complex, (**F**) PfProRS-SANC318 complex, (**G**) PfProRS-SANC184 complex, (**H**) PfProRS-SANC257 complex, (**I**) PfProRS-SANC264 complex, (**J**) PfProRS-SANC456 complex and (**K**) PfProRS-SANC622 complex.

Free energy landscapes show protein stability in terms of Gibbs free energy [58]. In this study, FEL of the holo protein and the ligand-bound complexes were analyzed using PCA, calculated over the 200 ns simulation, and plotted as 3D graphs with PC1 and PC2 displayed as a contour map at the bottom of each FEL plot to show different conformational states (Figure 6). Free energy landscape calculations showed that the holo system had three energy minima during the 200 ns simulation (Figure 6A). For the orthosteric ligands, the PfProRS-SANC152 system was the most stable with few intermediate states (Figure 6B). The PfProRS-SANC152 backbone RMSD distribution calculations also showed a normal distribution and a decrease in conformational flexibility (Figure 4B). PfProRS-SANC235 was the least stable complex as shown by the many intermediate states and only one with low energy basin (Figure 6C). PfProRS-SANC235 complex showed a multimodal backbone RMSD distribution augmenting the observation that this complex was the least stable of the orthosteric bound complexes (Figure 5D). PfProRS-SANC236, PfProRS-SANC244, and PfProRS-SANC318 systems showed four, three, and four main basins, respectively, with a total of three energy minima each (Figure 6D–F). These complexes showed several energy intermediate states during the 200 ns simulation implying they explored several unstable conformations and thus take longer time scales to reach their stable states.

PfProRS-SANC184 was the most stable complex among the allosteric ligand-bound systems as shown by the low energy with very few intermediates and hardly distinguishable low energy barriers (Figure 6G). The complex showed two low energy basins which is agreement with the bimodal backbone RMSD distribution observed during the 200 ns simulations (Figure 6G). PfProRS-SANC257 showed four main basins and five energy minima (Figure 6G). PfProRS-SANC257, PfProRS-SANC264, PfProRS-SANC456 and PfProRS-SANC622 showed more intermediate states and multiple lower free-energy barriers compared to PfProRS-SANC184 (Figure 6G–K). Backbone RMSD distribution calculations showed multimodal distributions for these complexes implying they explored several conformations during the 200 ns simulations (Figure 5G–K). These three complexes thus take longer time scales to reach their stable states due to the presence of multiple intermediate conformations.

**Figure 7:**
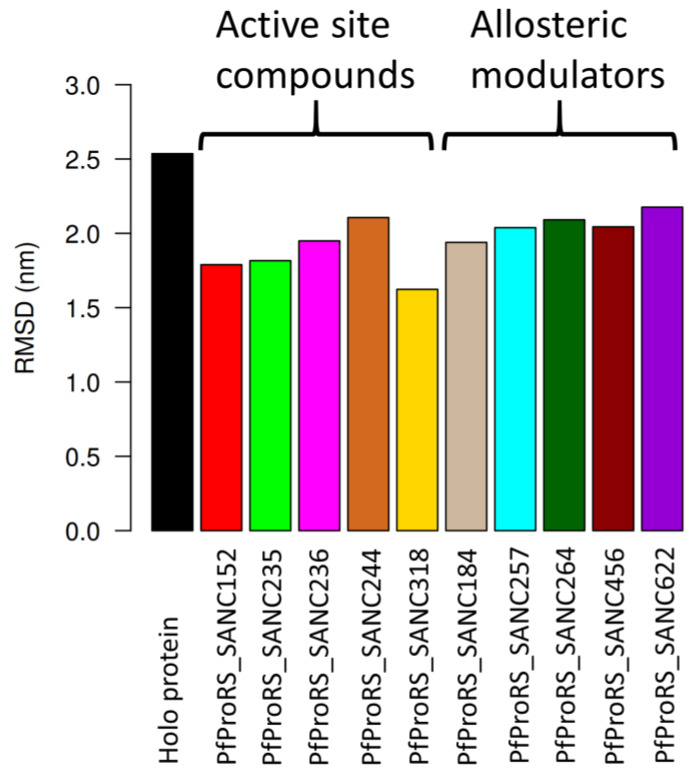
Bar graph representation of protein backbone root mean square deviation of FEL stable states compared to initial structures. The holo protein and protein-ligand complexes are shown on the X-axis.

Structures for the holo protein and ligand-bound complexes were obtained from the regions with low energy (stable states). These structures were then superimposed to the initial structure and the RMSD of each pair determined (Figure 7). Generally, orthosteric ligand-bound complexes showed lower RMSD values compared to allosteric ligand-bound complexes. Interestingly, PfProRS-SANC152 complex showed a low RMSD value of 1.79 Å, which supports the FEL results which showed this was the most stable orthosteric ligand-bound complex (Figure 7). On the other hand, PfProRS-SANC244 had the highest RMSD value of 2.11 Å explaining why this complex showed more intermediate states and the diverse conformations it explored as illustrated in PCA calculations (Figure S9E, Figure 6E and Figure 7). The RMSD values of PfProRS-SANC235, PfProRS-SANC236 and PfProRS-SANC318 compared to the initial structures were 1.82 Å, 1.95 Å and 1.62 Å respectively (Figure 7). For the allosteric ligand-bound complexes, PfProRS-SANC184 complex showed the lowest RMSD value of 1.94 Å while the RMSD values for PfProRS-SANC257, PfProRS-SANC264, PfProRS-SANC456, and PfProRS-SANC622 were 2.04 Å, 2.09 Å, 2.04 Å, and 2.18 Å, respectively (Figure 7). The RMSD of the holo protein was 2.54 Å which implies that the selected orthosteric and allosteric modulators slightly reduced the protein backbone RMSD. The low RMSD value of the PfProRS-SANC184 system may explain why this complex showed low energy across PC1 and PC2 among allosteric complexes as shown by FEL calculations (Figure 6G).

### 2.4. Ligand Binding Modulates Local Protein Motions

To understand the effect of ligand binding on local protein motions, per residue RMSF, DRN, evolution of hydrogen bond over the simulation time, and residue contribution to PC1 and PC2 for each ligand-bound complex were calculated. Root mean square flactuation calculations show the effect of ligand binding on residue flexibility while DRN analysis gives insights on residue connectivity which helps in understanding protein communication and function. Calculation of the residue contribution to PC1 and PC2 provides an understanding on which regions contribute most to the protein motions.

#### 2.4.1. Ligand Binding Modulates Residue Flexibility

**Figure 8:**
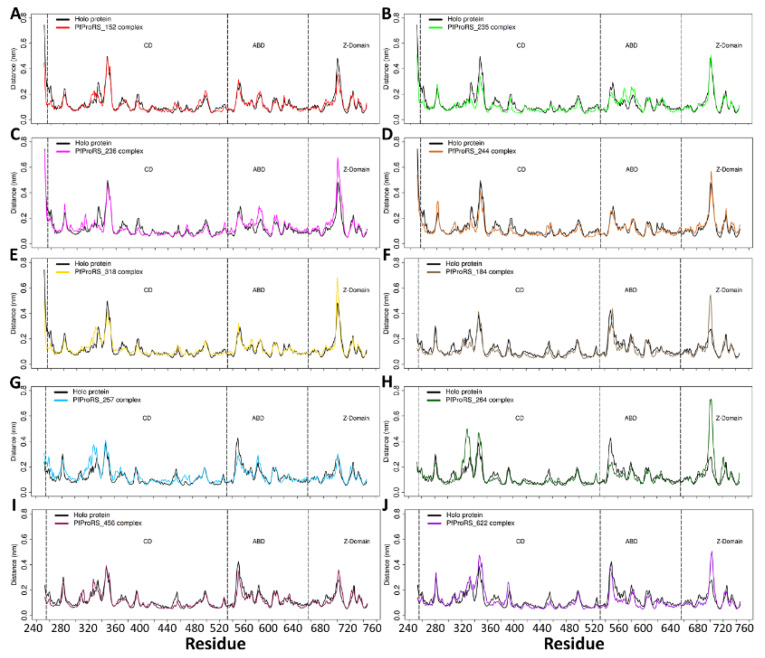
Per residue root mean square fluctuation analysis of the holo system and PfProRS ligand-complexes during the 200 ns simulation. (**A**) PfProRS-SANC152 complex, (**B**) PfProRS-SANC235 complex, (**C**) PfProRS-SANC236 complex, (**D**) PfProRS-SANC244 complex, (**E**) PfProRS-SANC318 complex, (**F**) PfProRS-SANC184 complex, (**G**) PfProRS-SANC257 complex, (**H**) PfProRS-SANC264 complex, (**I**) PfProRS-SANC456 complex and (**J**) PfProRS-SANC622 complex.

Generally, RMSF calculations for all the orthosteric and allosteric ligand-bound systems showed high peaks between residues 340–358, 540–560, and 688–720 which are loop regions (Figure 8). In PfProRS-SANC152 complex, ligand binding decreased the flexibility of residues 330–340, 350–380 and 390–405 (Figure 8A). There was a significant decrease in flexibility of residues 330–410 for the PfProRS-SANC235 complex compared to the holo protein (Figure 8B). Residues 386–405 form a loop at the active site whose movement changes the conformation of the active site and affects binding of ATP [29]. RMSF calculations of PfProRS-SANC236 showed a slight increase in flexibility of residues 560–590 and 695–715 of the ligand bound complex compared to the holo protein (Figure 8C). Residues 695–715 are part of two α-helices that are linked by a loop region at the Z-domain and comprise motif I that is involved in ATP binding [25,59].

Generally, all the allosteric ligand-bound systems showed an increase in residue flexibility between 320 and 360 in the protein-ligand complexes except for PfProRS-SANC184 complex (Figure 8F–J). These residues are part of a poorly resolved region (residues 316–351) in the available PfProRS crystal structures located at the dimer interface [34,60]. Interestingly, these residues showed a decrease in flexibility upon binding of the orthosteric ligands (Figure 8A–E). Most of the residues in PfProRS-SANC184 complex showed slight decrease in flexibility indicating that the ligand restricted movement of residues (Figure 8F). A significant increase in fluctuation was seen for residues 698–710 which form part of motif I [25,59] for PfProRS-SANC184, PfProRS-SANC264 and PfProRS-SANC622 complexes (Figure 8F,H,J). The flexibility of these residues may be related to the function of this region which is involved in binding of ATP [59]. Interestingly, this region did not show increase in residue flexibility upon binding of orthosteric ligands except for PfProRS-SANC236 complex (Figure 8A–E). Residues 540–570 which are part of the ABD showed a decrease in flexibility in PfProRS-SANC257 and PfProRS-SANC264 complexes as compared to the holo system (Figure 8G,H). There was no significant change in residue flexibility between the holo protein and PfProRS-SANC456 complex (Figure 8I). An increase in fluctuation of residues 320–360 and 698–710 upon binding of allosteric ligands may affect binding of substrates at the active site as these residues are implicated in binding of ATP and proline substrate [37]. In addition, in the allosteric bound complexes, residues implicated in orthosteric ligand binding showed slight increase in flexibility compared to orthosteric bound complexes (Figure 8F–J). These changes in dynamics of active site residues as a result of allosteric modulation may result in a decreased binding affinity of ProRS substrates.

RMSF calculations of PfProRS in complex with glyburide inhibitor showed a slight increase in flexibility of residues 330–360 at the CD and 690–710 in the Z-domain (Figure S10A). PfProRS-glyburide and PfProRS-halofuginone complexes showed a decrease in flexibility of most of the residues in the CD (Figure S10A,B). Notably, residues 690–710 in PfProRS-halofuginone complex showed a slight decrease in flexibility.

The effect of the stable ligands on HsProRS residue flexibility was also studied. In all HsProRS ligand complexes the was a decrease in flexibility of residues 1315–1330 at the ABD (Figure S11). In HsProRS-SANC152, HsProRS-SANC184 and HsProRS-SANC236 complexes, ligand binding increased the flexibility of residues 1075–1105 at the CD (Supplementary Figure S11A–C). In HsProRS-SANC257, ligand binding increased flexibility of residues 1450–1475 at the Z-domain (Supplementary Figure S11E).

#### 2.4.2. Evolution of Hydrogen Bond Interactions over the 200 ns Simulations

Hydrogen bond interaction between the protein and the ligand contributes to ligand stability. Hydrogen bond analysis for each ligand-bound complexes was done using the 200 ns trajectories to identify the residues involved in hydrogen bond interaction over the 200 ns simulation. PfProRS-SANC152 complex showed an average of four hydrogen bond interactions with only two of these bonds being consistent across the simulation (Supplementary Figure S12A). Gly455 contributed to the hydrogen bond interactions consistent across the simulation (Supplementary Figure S9A). On average, PfProRS-SANC235 formed two hydrogen bonds during the simulation (Supplementary Figure S8B). Further analysis of hydrogen bond interactions showed that residues Glu452, Phe454, and Lys453 contributed to these interactions (Supplementary Figure S12B). PfProRS-SANC236 complex had average of three hydrogen bonds which were contributed by residues Gly455, Ile332 and His331 (Figures S12C and S13C). A total of five hydrogen bonds were observed in PfProRS-SANC244 complex which were contributed by residues Ile332, His331, Arg401, Lys394 and Asn330 (Supplementary Figures S12D and S13D). Hydrogen analysis showed that PfProRS-SANC318 complex had three hydrogen bond interactions across the simulation which were contributed by Arg390, Glu351 and Ile332 (Supplementary Figures S12E and S13E).

Analysis of hydrogen bonds for PfProRS-SANC184 and PfProRS-SANC257 complexes showed a total of six bonds with only four of these bonds being consistent throughout the MD simulation (Figure S12F,G). Further analysis of hydrogen bond interactions in PfProRS-SANC184 showed that residues Pro396, Thr397, Ser263, Tyr278, Tyr285, Thr267, and Gln395 contributed to the hydrogen bond interactions (Supplementary Figure S14A). Residues Asn470, Tyr746, Ser263, Asp264, Ser708, and Lys394 contributed to the hydrogen bond interaction in PfProRS-SANC257 (Supplementary Figure S14B). PfProRS-SANC264 and PfProRS-SANC622 complexes made four hydrogen bond interactions with only two of these interactions being consistent throughout the 200 ns simulation (Figure S12H,J). In PfProRS-SANC264 system, Ser263, Leu707, Arg472, and Arg744 contributed to hydrogen bond interactions (Supplementary Figure S14C). Ser263, Lys394, Thr706, Leu707, and Tyr746 contributed to the hydrogen bond interactions in the PfProRS-SANC456 complex (Supplementary Figure S14D). Four hydrogen bond interactions were observed for PfProRS-SANC622 complex throughout the simulation which were contributed by Ser263, Gln395, Arg401, and Thr706 (Supplementary Figures S12E and S14J).

#### 2.4.3. Residue Contribution to PC1 and PC2 Motions

**Figure 9:**
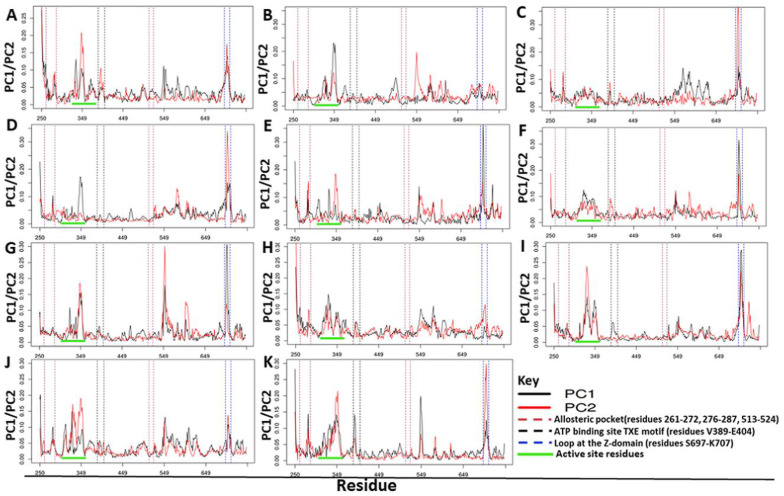
Contribution of each residue to PC1 and PC2. PC1 is shown in black while PC2 is shown in red for all the ligand-bound complexes. (**A**) Holo protein, (**B**) PfProRS-SANC152 complex, (**C**) PfProRS-SANC235 complex, (**D**) PfProRS-SANC236 complex, (**E**) PfProRS-SANC244 complex, (**F**) PfProRS-SANC318 complex, (**G**) PfProRS-SANC184 complex, (**H**) PfProRS-SANC257 complex, (**I**) PfProRS-SANC264 complex, (**J**) PfProRS-SANC456 complex and (**K**) PfProRS-SANC622 complex.

Further, to understand the effect of ligand binding on local protein motions, the contribution of each residue to PC1 and PC2 was analyzed (Figure 9). Generally, loop regions contributed most to motions during the 200 ns simulations. A loop region at the CD between residues 300–350 showed significant contribution to both PC1 and PC2 (Figure 9). The ABD also contributed significantly to the protein motions as seen at residues 540–570 that consists of α-helix and β-sheets linked by loops, an observation which agreed with RMSF calculations (Figure 8 and Figure 9). In all complexes, the loop region at the Z-domain (residues 697–710) showed high contribution to the motions represented by PC1 than PC2 except for PfProRS-SANC152 complex (Figure 9). Interestingly, the ATP binding TXE loop (residues 389–405) contributed more to motions represented by PC1 and PC2 for the allosteric complexes than for orthosteric complexes (Figure 9). The predicted allosteric site residues did not contribute significantly to large motions in regard to PC1 and PC2 probably because this region was stabilized by the binding of allosteric modulators (Figure 9). However, in allosteric ligand complexes, residues at the active site showed higher contributions to protein motions as described by PC1 and PC2 compared to orthosteric ligand complexes (Figure 9). Concomitantly, active site residues showed higher fluctuations in allosteric bound complexes compared to orthosteric bound complexes as shown by RMSF calculations (Figure 8). This implies that orthosteric ligands stabilize the active site residues upon binding as these changes in fluctuations were comparable to the holo protein system.

### 2.5. Ligand Binding Modulates Protein Communication

Based on the theory of residue centrality, highly connected nodes can be used to identify evolutionary conserved (central) residues important in protein function [61,62]. Further, residue interaction networks can be used to study various biological processes like catalytic activity, allosteric communication, mutation effects, protein intra- and inter-domain communications and protein folding [56,62,63,64]. Residue networks present the data in 2D thus reducing the complexity of 3D protein structures and enables the user to focus on individual amino acids and their interactions [64]. Residue networks have also been shown to be effective in studying the effect of residue changes for protein-ligand complexes [56,64,65,66,67].

Here, the effect of ligand binding on residue connectivity and protein communication was studied through DRNs. Dynamic residue networks were calculated for all MD simulation trajectories for only the Cα and Cβ atoms. Betweenness centrality (*BC*) and average shortest path (*L*) for all the ligand-bound systems were analyzed and compared to the holo protein via Δ*L* and Δ*BC* calculations. *Betweenness centrality* is a measure of how key a residue is to the protein communication. Residues with a negative Δ*BC* (average *BC* of holo protein minus average *BC* of protein-ligand complex) mean they gain more central role in protein communication in the ligand-bound complex while a positive Δ*L* (average *L* of holo protein minus average *L* of protein-ligand complex) means residues are more accessible in the ligand-bound complexes.

For the comparison between average *BC*, average *L* and RMSF all values were normalized as described in the methodology section. Pairwise Pearson’s correlation coefficient was calculated to establish the relationship between the three metrics (Table S3). In all the complexes average *BC* showed a negative Pearson’s correlation coefficient to RMSF (≤−0.36) (Supplementary Table S3). The difference in average *BC* (Δ*BC*) and average *L* (Δ*L*) between the holo protein and each ligand-bound complex was calculated to further analyze the effect of ligand binding on residue accessibility and protein communication.

**Figure 10:**
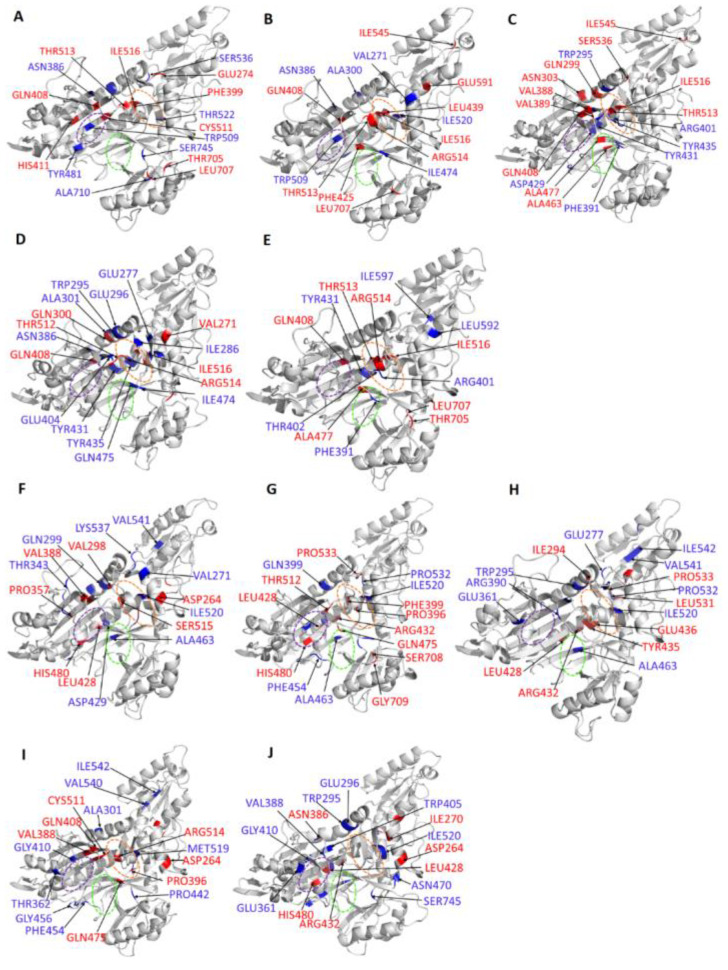
Cartoon representation of PfProRS homology model showing structural mapping of residues with significant Δ*BC* (2 standard deviations). Δ*BC* values were calculated by taking the *BC* value of the holo protein less the ligand-bound complex. Structures were obtained at 200 ns of the MD simulations. Residues with negative Δ*BC* values are shown in blue while residues with positive Δ*BC* values are shown in red. The active site is shown in green ellipses, ATP binding site in yellow ellipses and the predicted allosteric site in orange ellipses. (**A**) PfProRS-SANC152 complex, (**B**) PfProRS-SANC235 complex, (**C**) PfProRS-SANC236 complex, (**D**) PfProRS-SANC244 complex, (**E**) PfProRS-SANC318 complex (**F**) PfProRS-SANC184 complex, (**G**) PfProRS-SANC257 complex, (**H**) PfProRS-SANC264 complex, (**I**) PfProRS-SANC456 complex and (**J**) PfProRS-SANC622 complex.

Generally, regions at the allosteric site showed a decrease in *BC* for all ligand-bound systems compared to the holo protein (Figure 10 and Supplementary Figure S15). Residues with significant changes in *BC* at two standard deviations (negative Δ*BC*) were identified and presented in Table 3. Further, residues with significant changes in *BC* were mapped to the structure. Most of the residues with significant changes in *BC* are located at or close to the active site, allosteric site or at a loop at the Z-domain implying these sites are key in PfProRS communication (Figure 10, Supplementary Figure S15 and Table 3). Generally, allosteric complexes showed more residues with significant changes in *BC* compared to orthosteric complexes (Table 3). Allosteric complexes showed fewer residues with positive Δ*BC* at the ATP binding site compared to orthosteric ligands (Figure 10F–J). This implies that residues at this site have less important function in the allosteric complexes. Further, DRN analysis for known PfProRS inhibitors was carried out (Supplementary Figure S16). PfProRS-glyburide complex showed an increase in *BC* (negative Δ*BC*) for residues 260–290 at the allosteric site whereas residues 390–405 which are part of the ATP binding site showed a decrease in *BC* (positive Δ*BC*). Interestingly, in PfProRS-TCMDC124506 complex the changes in these regions were not significant. Residues 260–290 showed a decrease in *BC* (positive Δ*BC*) for PfProRS-halofuginone complex.

Further, analysis of *BC* and average *L* of the stable HsProRS complexes (HsProRS-SANC152, HsProRS-SANC184, HsProRS-SANC236, HsProRS-SANC244 and HsProRS-SANC257) were performed (Figures S17 and S19). In PfProRS complexes, residues 261–272 in the allosteric region showed a positive Δ*BC* while the corresponding region in HsProRS (residues 1023–1033) had a negative Δ*BC* (Supplementary Figures S15 and S17). Similarly, ligand binding in PfProRS resulted in a positive Δ*BC* of residues 276–287 which are part of the predicted allosteric site ligand binding while the corresponding residues (1039–1049) in HsProRS complexes showed a negative Δ*BC* (Figures S15 and S17). The decrease in *BC* (positive Δ*BC*) for PfProRS ligand complexes implies that these residues at the predicted allosteric site in PfProRS are probably losing the important function in HsProRS complexes, the negative Δ*BC* in this region implies that these residues may be gaining important function upon ligand binding. On the contrary, residues 293–313 in PfProRS complexes showed negative Δ*BC* while the corresponding region (residues 1055–1075) in HsProRS complexes showed positive Δ*BC* (Supplementary Figures S15 and S17). Similarly, in PfProRS, residues 513–524 in the predicted allosteric site showed negative Δ*BC* implying that these residues are gaining important function while the corresponding region (residues 1152–1169) in HsProRS resulted to positive Δ*BC* upon ligand binding meaning these residues in HsProRS are probably losing the important function (Figures S15 and S17). The loop region in the Z-domain (residues 697–705) showed higher positive Δ*BC* values in PfProRS ligand complexes compared with the corresponding region (Supplementary Figures S15 and S17) in HsProRS complexes implying that residues in PfProRS Z-domain which are implicated in ATP binding are losing the important function. Notably, sequence alignment results showed a deletion at the loop region in Z-domain of mammalian sequences and not in *Plasmodium* sequences (Supplementary Figure S2).

**Figure 11:**
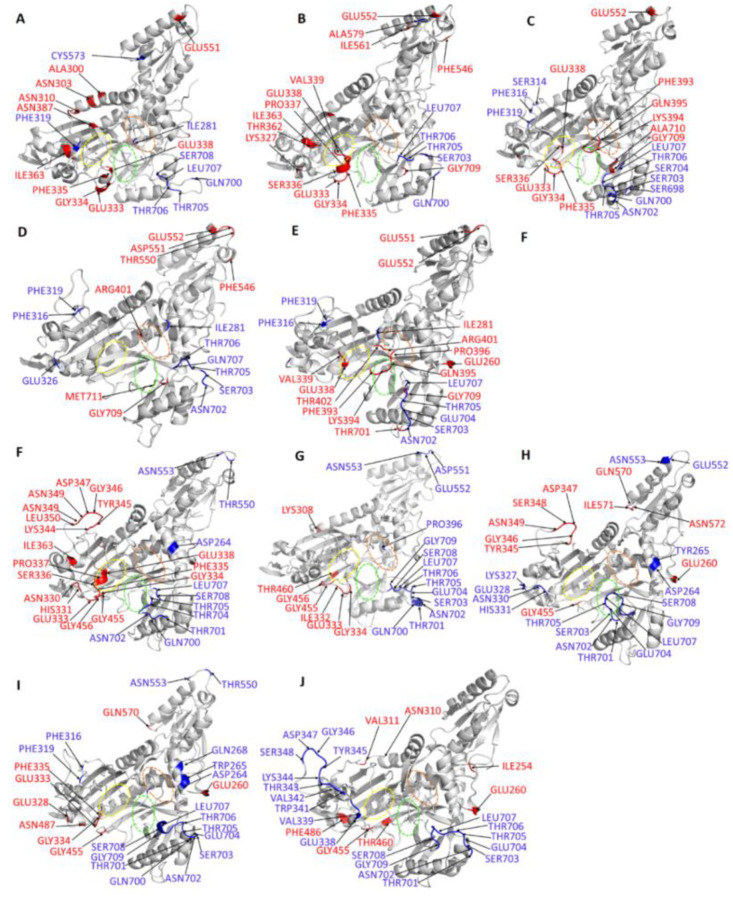
Cartoon representation of PfProRS homology model showing structural mapping of residues with significant Δ*L* (2 standard deviations). Δ*L* values were calculated by taking the *L* value of the holo protein less the ligand-bound complex. Structures were obtained at 200 ns of the MD simulations. Residues with negative Δ*L* values are shown in blue while residues with positive Δ*L* values are shown in red. The active site is shown in green ellipses, ATP binding site in yellow ellipses and the predicted allosteric site in orange ellipses. (**A**) PfProRS-SANC152 complex, (**B**) PfProRS-SANC235 complex, (**C**) PfProRS-SANC236 complex, (**D**) PfProRS-SANC244 complex, (**E**) PfProRS-SANC318 complex (**F**) PfProRS-SANC184 complex, (**G**) PfProRS-SANC257 complex, (**H**), PfProRS-SANC264 complex, (**I**) PfProRS-SANC456 complex and (**J**) PfProRS-SANC622 complex.

Average *L* showed a positive correlation to the residue RMS fluctuation calculations with high Pearson’s correlation coefficients (≥0.54) (Supplementary Table S3). Residues with significant changes in average *L* are shown in Table 2. For all the systems average *BC* and inverse *L* showed a positive Pearson’s correlation coefficient (≥0.71) (Supplementary Table S3). The positive correlation between *BC* and inverse *L* was shown for the first time by Penkler et al. [56]. Residues 320–330, 540–550, and 695–710 showed a negative Δ*L* after ligand binding as shown in Δ*L* calculations indicating that ligand binding decreased accessibility of these regions (Supplementary Figure S18, Table 4). Residues 330–360, 450–470 and 570–585 showed significant decrease in average *L* upon ligand binding which implies that ligand binding increases connectivity of these residues (Figure 11 and Supplementary Figure S18). Although not all the residues in these regions have been reported in ligand binding, previous studies have shown that ligand binding can affect accessibility of residues at locations distant from the binding site [56]. Interestingly, in all ligand-bound complexes, residues 261–287 and 513–524 which form the predicted allosteric pocket had a negative Δ*L* implying that binding of ligands decreased accessibility of residues at this site (Supplementary Figure S18). Furthermore, residues at a loop (697–710) in the Z-domain showed a negative Δ*L* but the effect was more pronounced for allosteric ligand complexes (Table 4, Figure 11 and Supplementary Figure S18). Decrease in accessibility of residues at this loop region in Z-domain may result to reduced ATP binding affinity as they are implicated in ATP binding. Despite the negligible changes in average *L* on residues implicated in ligand interactions, the deep tunnels observed at the binding sites signify high accessibility of residues at these regions. Changes in accessibility of residues at the active site, allosteric pocket and the Z-domain signify the importance of these regions in protein communications. PfProRS-glyburide, PfProRS-halofuginone and PfProRS-TCMDC124506 complexes showed an increase in *L* (negative Δ*L*) in a loop region consisting of residues 690–710 at the Z-domain (Figure S16). PfProRS-glyburide and PfProRS-TCMDC124506 complexes showed an increase in average *L* (negative Δ*L*) in the ATP binding domain (residues 390–405) whereas PfProRS-halofuginone complex showed a decrease in *L* for these residues (Supplementary Figure S16), which was in line with the observations made for the selected hit compounds (Supplementary Figure S18).

On carrying out average *L* analysis, a region in the allosteric site consisting of residues 261–286 in PfProRS and the corresponding region (residues 1025–1048) in HsProRS showed a negative Δ*L* and complexes (Figures S18 and S19). Interestingly, a loop region (residues 389–405) implicated in ATP binding in PfProRS showed negative Δ*L* while the corresponding region (residues 1152–1169) in HsProRS complexes showed a positive Δ*L* (Supplementary Figures S18 and S19). This implies that, in HsProRS complexes, there is increased accessibility of residues at the ATP binding loop compared to the corresponding residues in PfProRS. In HsProRS-SANC184, HsProRS-SANC257 and HsProRS-SANC318 complexes, residues 1464–1473 in a loop region in the Z-domain corresponding to residues 697–707 in PfProRS showed positive Δ*L* implying there is increased accessibility of these residues while in HsProRS-SANC152 and HsProRS-SANC244 complexes, these residues showed negative Δ*L* (Figure S18). In PfProRS complexes, residues 513–524 in the allosteric site and the corresponding region in HsProRS (residues 1277–1287) had no significant change in average *L* (Supplementary Figures S18 and S19).

#### Allosteric Modulators Increase Contact Frequency between Allosteric and ATP Binding Site Residues

**Figure 12:**
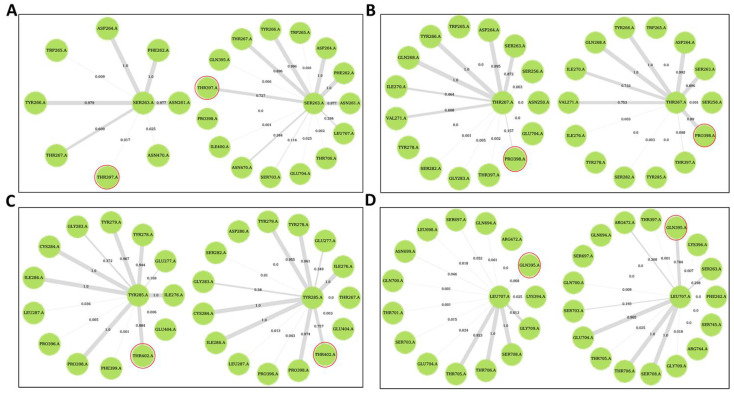
Frequency of contacts at the determined allosteric site in the holo (left sub-figure) and the ligand-bound complexes (right sub-figure). The allosteric site residue is shown in the middle of each contact map while residues at the ATP binding TXE loop are circled in red. (**A**) Contact frequency between residue Ser263 and Thr397 (**B**) Contact frequency between residue Thr267 and Pro398, (**C**) Contact frequency between residue Tyr285 and Thr402 and (**D**) Contact frequency between residue Leu707 and Gln395.

Contact map calculation outputs RINs in two dimensions thus enabling the study of interactions between domains or secondary structural elements [63]. To study how residues at the ATP binding site are affected by the allosteric site ligands, contact maps were calculated for the residues interacting with the selected allosteric modulators at the postulated allosteric site, and the residues with significant increase in contact with residues compared to the holo protein residues were selected. Among these, Ser263 involved in binding of SANC184 and SANC264, Thr267 (SANC264), Tyr285 (SANC184), and Leu707 (SANC184 and SANC456) showed significant increase in frequency in the interaction to residues Thr397, Pro398, Thr402 and Gln395 respectively which are all part of the ATP binding TXE loop (Figure 12). An increase in the interaction frequency of these residues at the ATP binding TXE loop upon allosteric ligand binding may explain why there is distortion of the ATP binding site after ligand binding at the allosteric site. In a previous study, Hewitt et al., 2017, reports that inhibition by allosteric ligands is through distortion of the ATP binding site through movement of the ATP binding TXE loop [29]. Betweenness centrality calculations also showed an increase in *BC* for the ligand-bound complexes implying that ligand binding increases communication of residues at the allosteric site with residues at the ATP binding site.

**Figure 13:**
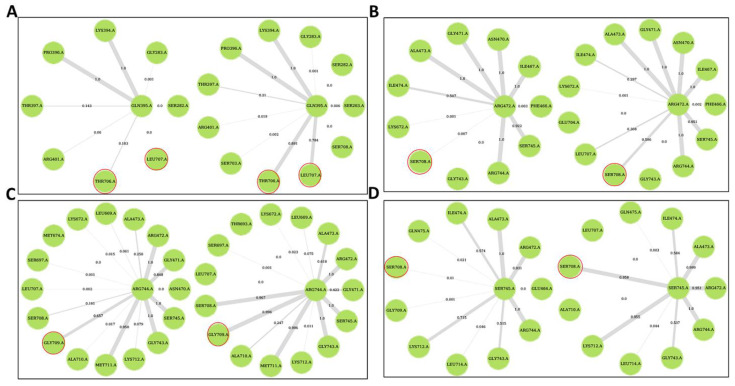
Frequency of contacts at the determined allosteric site in the holo (left sub-figure) and the ligand-bound complexes (right sub-figure). The allosteric site residue is shown in the middle of each contact map while residues at the Z-domain are circled in red. (**A**) Contact frequency between Gln395, Thr706 and Leu707, (**B**) Contact frequency between Arg472 and Ser708, (**C**) Contact frequency between Arg744 and Gly709 and (**D**) Contact frequency between Ser745 and Ser708.

RMSF and DRN calculations showed significant changes in a loop at the Z-domain upon ligand binding, thus we also analyzed the contact frequency between residues implicated in ligand binding and residues in this loop. Gln395 implicated in binding of SANC622, Arg472 (SANC257, SANC264 and SANC456), Arg744 (SANC264), and Ser745 (SANC456) also showed an increase in interaction frequency with residues Thr706, Leu707, Leu708, Gly709 and Ser708 respectively (Figure 13). Residues Thr706, Leu707, Leu708 and Gly709 are located in the Z-domain region which was characterized by large motions in all ligand-protein complexes. Δ*BC* calculations showed that residues Val697, Pro713, Ser703, Gln700, Gly743 and Ser745 located in the Z-domain which are implicated in ATP binding gained importance in the allosteric complexes implying allosteric ligands may affect ATP binding affinity. Residues in a loop region (700–710) had negative Δ*L* values in all complexes, implying ligand binding may result to decrease in accessibility in this region. This region was also characterized by high residue fluctuations and large motions during MD simulations as shown by RMSF and PCA calculations. These changes in protein motions and accessibility of the Z-domain may affect ATP affinity as residues in this region are implicated in ATP binding.

### 2.6. Selected Allosteric Modulators Affect PfProRS Function through Distortion of the ATP Binding Site

Allostery is the process by which perturbing or binding of a ligand at a specific site in a protein results to a long-range change in the chemical and/or physical properties of another, often a functional site of the protein [68,69,70,71]. Allostery is important in understanding disease, cellular signaling, drug design and molecular mechanisms of proteins [68,69,72,73]. In this study, allostery was studied through MD, FEL, DRNs, and weighted contact maps of protein-ligand complexes.

The changes that were seen in RMSF and DRN calculations at the ATP binding site upon binding of ligands at the predicted allosteric site may result to possible distortion of the ATP binding site. In a previous study, binding of glyburide and TCMDC-124506 inhibitors at the postulated allosteric site was shown to distort the ATP binding site [29]. Glyburide and TCMDC-124506 inhibitor binding at the allosteric site causes a shift of a loop consisting of residues 389 to 404 thus displacing key residues involved in ATP binding [29]. Arg390 forms hydrogen bond interactions with the α and β phosphates, Phe405 forms π-π stack interactions with the adenosine ring while Arg401 interacts with the γ-phosphate of nucleotides through a hydrogen bond interaction [29]. The binding of allosteric ligands to residues close to the ATP binding site may cause shifting of the C-terminal end of the loop towards the nucleotide binding site hence causing distortion of the pocket.

## 3. Methodology

### 3.1. Data Retrieval

*Plasmodium falciparum* ProRS sequence (accession number: Q8I55R7) was retrieved from National Centre for Biotechnology Information (NCBI) protein database [74]. Other *Plasmodium* and mammalian homologues including HsProRS were searched in UniProt and NCBI using PfProRS as the query sequence, BLASTp algorithm and the BLOSUM62 matrix [75,76,77]. Fifteen sequences in total (8 plasmodial and 7 mammalian) were retrieved (see Supplementary Table S1). Crystal structures for *P. falciparum* and human ProRS (PDB ID: 4WI1 [29], 4K88 [33], and 4HVC [44], respectively) were retrieved from PDB [78].

### 3.2. Sequence Alignment

Multiple sequence alignment (MSA) of the retrieved sequences was carried out using MUltiple Sequence Comparison by Log-Expectation (MUSCLE), Tree-based Consistency Objective Function Evaluation (TCOFFEE) and Profile Multiple Alignment with Local Structures and 3D constraints (PROMALS3D) alignment tools [79,80,81,82]. Visualization of the alignment was done using the Jalview vs. 2.10 software [83]. MUSCLE program gave good alignment at conserved regions with inserts only occurring in loops and thus it was considered as the best alignment to use in further analyses.

### 3.3. Homology Modelling

The ProRS crystal structures have missing residues, thus homology models were used to build the missing residues via MODELLER [84]. Residues 241, 280–282, 322–359, and 389–397 were missing in PfProRS crystal structure 4WI1 [29]. HsProRS crystal structure 4K88 was missing residues 1001–1015 at the N-terminal and 1465–1473 [33]. For the modelling of PfProRS, 4HVC (HsProRS) [44] and 4WI1 (PfProRS) [29] crystal structures were used as templates, while 4K88 [33] was used to remodel HsProRS excluding the missing residues at the N-terminal (1001–1015). PfProRS was also modelled with known inhibitors; glyburide, halofuginone and TCMDC-124506. For halofuginone, 4YDQ [37]; for modelling PfProRS with glyburide, 5IFU [29]; for TCMDC-124506, 4WI1 [29] was used. Templates were identified using HHpred [85] and PRotein Interactive MOdeling (PRIMO) webservers [86]. For each protein, 100 models were calculated by MODELLER, and the top three models with the lowest z-DOPE (Discrete Optimized Protein Energy) score were selected for validation. Further, structure quality assessment was done using qualitative model energy analysis (QMEAN) [87], Protein Structure Analysis (PROSA) [88] and Verify3D webserver [89] and the model with the best scores was selected [25] for molecular docking and subsequent calculations.

### 3.4. Molecular Docking

A total of 623 minimized ligands were retrieved from SANCDB [45] for high throughput virtual screening. Ligands and homology models were prepared using AutoDockTools (ADT) [90] for the docking simulations. Partial atomic charges of the compounds and homology models were assigned using Gasteiger–Huchel protocol in AutoDock [90]. In this study, a Python script was used to calculate the box size and box center of PfProRS and HsProRS. A box size of 69.42 Å, 80.83 Å, 67.50 Å with box center of −5.53 Å, 15.30 Å, and 58.78 Å for the x, y, and z axes, respectively, was used for PfProRS. For the HsProRS docking, a box size of 77.47 Å, 93.09 Å and 66.11 Å was used while the center was set at 39.20 Å, 5.81 Å, and −7.05 Å for the x, y, and z coordinates, respectively. For the docking experiments, the ligands were allowed to be flexible while the protein was kept rigid during the docking process and screening was done for the entire protein surface (blind docking). To validate the docking studies, the co-crystallized ligand- TCMDC-1245061 in PfProRS (PDB ID: 4WI1) was redocked. The interactions between the best redocked pose and the co-crystallized ligand, and the protein were then analyzed using LigPlot^+^ tool [91]. Further, the RMSD of the heavy atoms of co-crystallized TCMDC-1245061 and the best redocked pose was determined. Protein-ligand docking was carried out in a Linux-based cluster using AutoDock Vina with adenosine (ADN) present at the ATP binding site [92]. The docking poses of all the ligands were visualized using PyMOL tool [93]. Ligands were then sorted based on the docking energies, and further based on whether the ligand was selectively binding to PfProRS and not to HsProRS active and/or allosteric sites. Discovery studio visualizer [94] was used to visually analyze docked ligands to identify interactions at the active site and allosteric site. Hydrophobic and hydrogen bond interactions between active site or allosteric site residues and each ligand were determined using Discovery Studio [94] and 2D maps for ligand interactions were generated. Lipinski’s rule of five (Ro5) was calculated using SCFBio webserver to determine the drug likeness of the compounds identified as hits [95,96].

### 3.5. Molecular Dynamic Simulations

The docking results with the lowest energy combinations for the SANCDB compound hits in complex with ProRS protein were used as starting structures for MD. Additionally, MDs for the PfProRS-known inhibitor complexes (glyburide and TCMDC-124506) were performed to study the effect of these inhibitors on the protein. Molecular dynamics simulations were carried out by GROMACS 2016.4 software [97]⁠ using the AMBER-03 force field [98]. ACPYPE tool was used to generate the SANCDB ligand topology files [99]⁠. The simple point charge (SPC) water model was used to solvate the holo protein (protein–ADN complex) and protein–ligand systems in a cubic box with the relative distance between the edge of the box and the molecules set at 1 nm [100]⁠. Na^+^ and Cl^-^ ions were added to neutralize the system. The system was subjected to energy minimization prior to production runs using a conjugate-gradient method with up to 50,000 steps and a steepest-descent algorithm and was terminated when a maximum force of <1000 kJ/mol was achieved to relax the structure [101]. Pressure and temperature equilibrations were done in two phases using the NPT (number of particles, pressure and temperature) and NVT (number of particles, volume and temperature) ensemble at 1 atm and 300 K respectively with each phase running for 100 ps, until an average pressure of 1 atm was achieved. The LINCS algorithm was used to constrain all interactions including hydrogen bond interactions [102,103]. The particle mesh Ewald algorithm was used to describe the long-range electrostatic interactions [104]. The barostat and thermostat coupling for each system was done using the Parrinello–Rahman and Nose–Hoover methods [105,106]. The simulation time step was set at 2 fs and periodic boundary conditions were applied in all directions. For each protein-ligand complex and the holo protein, the simulation was carried out for 200 ns. The root-mean square fluctuations (RMSF) of the C-alpha atoms, root mean square deviation (RMSD) with respect to the initial protein backbone structure, hydrogen bond distribution and radius of gyration for each system were determined. The RMSD with respect to the internal protein energy and the initial structures was used to check if the system had equilibrated. All MD simulations were carried out using a Linux-based cluster at the Centre for High Performance Computing (CHPC) at Cape Town, South Africa. Structures for analysis of ligand binding interactions were generated at 200 ns of the MD simulation and PyMol and Discovery Studio programs were used for visualization and analysis. All MD graphs were generated using an in-house R-script. Residues involved in hydrogen bond interactions were analyzed using Cpptraj tool and graphs generated using gnuplot [107].

### 3.6. Dynamic Residue Network Analysis

To study inter- and intra-domain communication changes over a time period as a result of ligand binding, dynamic residue network (DRN) analysis was performed. Dynamic residue network was applied to the holo system and the ligand-bound complexes. Residue network analysis is a graphical representation of protein structures in which amino acids are represented as nodes and the non-covalent interactions between the residues are referred as edges [108]. For each MD trajectory, residue networks were constructed by treating C_β_ atoms (C_α_ for glycine) as nodes in the network and connections between nodes established based on a distance cut-off of 6.7 Å. The resultant DRNs analyzed via two metrics: average long-range residue reachability (*L*) and average betweenness centrality (*BC*). The metric *L* is defined as the number of connections required to reach residue i from j using the shortest possible path. The average reachability of a residue (*Li*) is thus defined as the average number of steps required to reach residue i from any other residue in the network. The average *L* was calculated by dividing all shortest paths to a given node by the total number of paths. The metric *BC* is a measure of how often on average a residue is utilized in shortest path navigation [56]. In this study, the *BC* and *L* of all the protein-complex systems were calculated using the calc_network.py in MD-TASK for every 100 th frame of each trajectory [108]. For comparison, RMSF *BC* and *L* data were normalized using the following equation:

$$x_{norm}=\frac{\left(x-x_{min}\right)}{\left(x_{max}-x_{min}\right)}$$

where *x_norm_* is the normalized value, *x* is a value in the *BC* or *L* matrix, *x_min_* is the minimum value in the matrix and *x_max_* is the maximum value in the matrix. Changes in *BC* and *L* (Δ*BC* and Δ*L*) in the ligand-bound complexes were calculated by taking each value in the holo protein *BC* or *L* matrix less the corresponding value in the ligand-bound complex *BC* or *L* matrix.

To determine how often a residue was interacting with nearby residues, weighted contact maps were generated for all the allosteric site residues implicated in ligand binding using contact_map.py script in MD-TASK [108].

### 3.7. Principal Component Analysis

Principal component analysis is a multivariate method that decomposes protein dynamics, and extracts the dominant modes to reduce the number of motions required to describe the protein dynamics [57,109]. The method uses a 3N × 3N covariance matrix to extract the functionally most important motions using the coordinates that describe the protein dynamics. A cartesian coordinate of PCA depends on the overall MD, and uses cartesian coordinates to describe the degrees of freedom of all the conformations in the trajectory [57]. In the cartesian coordinate space, elements of the covariance matrix (C) are defined as

$$C_{ij}=\langle (x_{i}-\langle x_{i}\rangle )\left(x_{j}-\langle x_{j}\rangle \right)\rangle$$

where the brackets show an average of all structures sampled across the MD trajectory. The covariance matrix eigenvalues are decomposed to eigenvectors (collective modes) each with a corresponding variance or eigenvalue that describes a part of the protein dynamics with the large eigenvectors describing the dominant motions of the protein [57]. In this study, the first five principal components were calculated using a Python script (Supplementary Table S2). The first and the second principal components (PC1 and PC2) were used to describe the protein motions of all atom MD simulation over the 200 ns trajectory and plots generated using a Python script. Further, the contribution of each residue to PC1 and PC2 was calculated by the bio3d function in R. Gibbs free energy as a function of PC1 and PC2 for the holo protein and ligand-bound complexes was calculated using gmx sham, sham.pl and xpm2txt.py tools in GROMACS [97]. Gibbs free energy is given by:

$$\Delta G=\sum -{\mathrm{KT}}_{B}ln\left(P_{A}-P_{B}\right)$$

In this equation, Gibb’s energy is defined by ΔG which is a function of the equilibrium constant (K) and the gas constant (T_B_). P_A_ and P_B_ are the probabilities of conformation A and conformation B of a protein occurring during the dynamic simulation [58]. The FEL plots were generated using an R script.

## 4. Conclusions

In summary, ten selective novel inhibitors that target the active site or the proposed allosteric site of PfProRS were identified in this study. Selected hits had good binding energy scores and a number of hydrogen bonds that contribute to the stability of protein-ligand complexes in both the active and predicted allosteric site. Residues at catalytic sites are highly conserved across homologous proteins thus designing drugs that inhibit these regions with less or no toxic effects to the host is quite challenging. On the other hand, allosteric sites are less conserved and thus present desirable targets for design of drugs with high specificity. In a previous study, Nyamai and Tastan Bishop identified PfProRS allosteric pockets using FTMap and SiteMap tools for inhibitor docking. This study sought to identify inhibitors against PfProRS active site and understand allosteric modulation of PfProRS through MD, PCA, FEL, DRN and contact map calculations. Ligand binding caused a change in conformation of the protein backbone in PfProRS-SANC184 complex as shown by the bimodal distribution of RMSD during the 200 ns compared to the normal distribution seen in other complexes. Interestingly, FEL calculations showed PfProRS-SANC184 complex as the most stable complex as indicated by low energy with hardly distinguishable low energy barriers and very few intermediates. All complexes showed high RMS fluctuations of residues at the allosteric site which was in line with DRN results that showed increase in *BC* at this region. The allosteric site region also showed low values of average *L* indicating high accessibility of this region. Interestingly, ligand binding caused a decrease in accessibility of residues at the Z-domain, but the effect was more pronounced in the allosteric ligand-bound complexes. Furthermore, allosteric modulators caused a change in dynamics of ATP binding site residues as shown by DRN calculations implying these ligands may affect ATP binding in PfProRS. In addition, contact map calculations of residues at the allosteric site showed increase in contact frequency with residues at the ATP binding TXE loop implying binding of ligands at this site may cause distortion of the ATP binding site. The scaffolds of the selected hits can be used as a starting point for development of antimalarial inhibitors with minimal human cytotoxicity.

## Figures and Tables

**Table 1:** South African natural compound hits and docking binding energy information.

Compound Information			Docking Binding Energy (kcal/mol)	
Code Name	SANCDB ID	Chemical Name	PfProRS	HsProRS
SANC152	SANC00152	Tsitsixenicin D	−9.6	−7.4
SANC235	SANC00235	Sodwanone A	−10.2	−8.2
SANC236	SANC00236	Aplysulphurin-1	−10.9	−8.2
SANC244	SANC00244	Eucomnalin	−9.2	−7.4
SANC318	SANC00318	Crinamine	−7.9	−7.1
SANC184	SANC00184	Latrunculin B	−8.3	−7.9
SANC257	SANC00257	20-Hydroxy-20-epi-tingenone	−9.4	−9.2
SANC264	SANC00264	Tingenone	−9.7	−9.4
SANC456	SANC00456	Gordonoside A	−8.3	−8.5
SANC622	SANC00622	Seneciphylline	−7.8	−8.0

**Table 2:** Mean RMSD of PfProRS and HsProRS ligand complexes and average percentage differences. Average % differences are calculated for holo protein minus protein-ligand complexes.

Protein Complex	RMSD Mean (nm)	% Difference (Holo Protein Less Ligand Complex)
PfProRS-ADN	0.30	0.00
PfProRS-SANC152	0.25	16.67
PfProRS-SANC235	0.25	16.67
PfProRS-SANC236	0.27	10.00
PfProRS-SANC244	0.27	10.00
PfProRS-SANC318	0.26	13.33
PfProRS-SANC184	0.29	3.33
PfProRS-SANC257	0.24	20.00
PfProRS-SANC264	0.34	−13.33
PfProRS-SANC456	0.21	30.00
PfProRS-SANC622	0.25	16.67
PfProRS-halofuginone	0.25	16.67
PfProRS-glyburide	0.28	6.67
PfProRS-TCMDC124506	0.23	23.33
HsProRS-ADN	0.20	0.00
HsProRS-SANC152	0.24	−20.00
HsProRS-SANC184	0.20	0.00
HsProRS-SANC236	0.31	−55.00
HsProRS-SANC244	0.24	−20.00
HsProRS-SSANC257	0.21	−4.50

**Table 3:** Residues with significant changes in average *BC* (2 standard deviations away). Residues at the active site are shown in green, residues at the ATP binding site in purple and residues at the Z-domain in magenta.

**Protein-Ligand Complex**	**Residues with Significant Increase in Average *BC* for the Orthosteric Ligand Complexes**	**Residues with Significant Decrease in Average *BC* for the Orthosteric Ligand Complexes**
PfProRS-SANC152	300, 745, 710, 299, 456, 386, 481, 536, 469, 362, 333, 509, 522, 454, 537	516, 408, 513, 707, 517, 274, 439, 514, 411, 705, 511, 389
PfProRS-SANC235	300, 386, 271, 509, 456, 520, 474, 425	516, 513, 707, 408, 514, 545, 544, 530, 591, 594, 477, 439
PfProRS-SANC236	431, 295, 435, 512, 406, 401, 440, 393, 402, 456, 429	408, 513, 516, 299, 463, 536, 477, 303, 388, 594, 545, 389
PfProRS-SANC244	401, 295, 296, 286, 386, 300, 435, 532, 475, 407, 520, 519, 431, 474, 277, 294, 461, 297, 404	516, 514, 517, 408, 513, 299, 271, 594, 707, 512
PfProRS-SANC318	474, 401, 402, 393, 431, 592, 597	408, 513, 516, 707, 477, 594, 596, 705, 319, 514
**Protein-Ligand Complex**	**Residues with Significant Increase in Average *BC* for the Allosteric Ligand Complexes**	**Residues with Significant Decrease in Average *BC* for the Allosteric Ligand Complexes**
PfProRS-SANC184	299, 463, 303, 456, 537, 312, 536, 271, 442, 357, 641, 453, 343, 540, 509, 429, 520	513, 516, 388, 358, 440, 264, 515, 617, 480, 407, 428, 298
PfProRS-SANC257	454, 532, 745, 520,299, 472, 456, 307, 509, 453, 463, 429, 481, 460, 540, 653, 657, 641	440, 708, 428, 432, 396, 475, 512, 399, 533, 617, 480, 593, 709, 270, 264, 436
PfProRS-SANC264	295, 463, 537, 520, 532, 542, 277, 410, 540, 390, 595, 405, 307, 577, 361	440, 593, 428, 533, 514, 530, 436, 596, 435, 294, 432, 524
PfProRS-SANC456	519, 745, 509, 454, 410, 442, 333, 456, 542, 362, 540, 300	514, 512, 511, 617, 264, 388, 432, 396, 593, 408, 708, 475
PfProRS-SANC622	463, 520, 389, 410, 296, 361, 295, 521, 458, 460, 407, 745, 385, 641, 317, 470, 442, 429	593, 428, 386, 264, 432, 396, 270, 617, 298, 531, 480

**Table 4:** Residues with significant changes in average *L* (2 standard deviations away). Residues at the active site are shown in green, residues at the ATP binding site in purple and residues at the Z-domain in magenta.

**Protein-Ligand Complex**	**Residues with Significant Decrease in Average *L*** **in the Orthosteric Ligand Complexes**	**Residues with Significant Increase in Average *L*** **in the Orthosteric Ligand Complexes**
PfProRS-SANC152	333, 260, 335, 363, 362, 310, 552, 308, 307, 334, 303, 386, 338, 311, 300, 309	705, 707, 706, 708, 281, 319, 699, 700, 573, 553, 349
PfProRS-SANC235	552, 709, 338, 334, 335, 333, 327, 386, 337, 363, 362, 546, 336, 339	706, 707, 705, 579, 703, 700
PfProRS-SANC236	552, 338, 333, 701, 339, 334, 335, 336, 337, 395, 709, 711, 332, 710, 393, 394	703,705, 707, 704, 706, 698, 319, 700, 702, 314, 316
PfProRS-SANC244	550, 551, 709, 552, 401, 711, 546	707, 706, 703, 705, 349, 702, 368, 347, 700, 704
PfProRS-SANC318	552, 709, 395, 260, 701, 396, 551, 393, 401, 338, 394, 259, 402, 339	705, 707, 703, 328, 704, 319, 316, 281, 702
**Protein-** **Ligand Complex**	**Residues with Significant Decrease in Average *L* in the Allosteric Ligand Complexes**	**Residues with Significant Increase in Average *L* in the Allosteric Ligand Complexes**
PfProRS-SANC184	333, 349, 335, 334, 345, 347, 338, 348, 343, 350, 310, 344, 455, 337, 336, 456, 363, 331, 346, 330, 311	704, 701, 705, 702, 703, 707, 553, 700, 708, 264, 550
PfProRS-SANC257	333, 455, 334, 456, 738, 332, 460, 457, 308	701, 702, 705, 708, 706, 704, 703, 709, 553, 552, 707,396, 700, 550
PfProRS-SANC264	349, 347, 348, 346, 455, 345, 260, 572, 571, 570	704, 705, 708, 702, 703, 709, 701, 328, 553, 327, 330, 331, 707, 706, 552, 264, 265
PfProRS-SANC456	260, 333, 334, 335, 328, 455, 485, 570	701, 708, 705, 702, 707, 706, 704, 709, 396, 316, 703, 553, 319, 550, 264, 265, 268, 700
PfProRS-SANC622	260, 455, 310, 308, 254, 311, 486, 460	701, 702, 704, 706, 707, 703, 341, 708, 339, 319, 705, 338, 709, 346, 347, 342, 348, 343, 345, 344

## Supplementary Materials

Supplementary materials can be found at https://www.mdpi.com/1422-0067/21/11/3803/s1.

## Abbreviations

CD	Catalytic Domain
ABD	Anticodon Binding Domain
NTD	N-terminal Domain; MD, Molecular Dynamics
DRN	Dynamic Residue Network
RMSD	Root mean square deviation
RMSF	Root mean square fluctuation
SANCDB	South African Natural Compound Database
MSA	Multiple sequence alignment
AND	Adenosine
PCA	Principal component analysis
FEL	Free energy landscape
aaRS	aminoacyl tRNA synthetase
ProRS	Prolyl tRNA synthetase
