# Supplementary material for: Identification of Selective Novel Hits against Plasmodium falciparum Prolyl tRNA Synthetase Active Site and a Predicted Allosteric Site Using In Silico Approaches
Source: Int J Mol Sci. 2020 May 27;21(11):3803. doi: 10.3390/ijms21113803 (PMC7312540; doi:10.3390/ijms21113803)
Supplement: Supplementary file 1 [file ijms-21-03803-s001.pdf]

Identification of Selective Novel Hits against *Plasmodium falciparum* Prolyl tRNA Synthetase Active Site and a Predicted Allosteric Site using *in silico* Approaches

Dorothy Wavinya Nyamai, Özlem Tastan Bishop*

Research Unit in Bioinformatics (RUBi), Department of Biochemistry and Microbiology, Rhodes University, Grahamstown 6140, South Africa

Dorothy W Nyamai: dornyam@gmail.com, ORCID ID: https://orcid.org/0000-0003-4704-9883

*Correspondence to Özlem Tastan Bishop o.tastanbishop@ru.ac.za

Table S1: Prolyl tRNA synthetase sequence details.

Organism	E-value	Identity (%)	Accession code	Coverage (%)
Plasmodium falciparum	----	100.0	Q8I5R7	100.0
Plasmodium malariae	0.0	80.6	SBS90690.1	100.0
Plasmodium ovale	0.0	78.1	SCQ16956.1	99.0
Plasmodium knowlesi	0.0	77.8	XP_002262215.1	99.0
Plasmodium yoelii	0.0	76.8	ETB61604.1	99.0
Plasmodium fragile	0.0	79.2	XP_012336932.1	98.0
Plasmodium vivax	0.0	78.5	KMZ90180.1	98.0
Plasmodium berghei	0.0	76.5	XP_677690.2	99.0
Homo sapiens	4e-173	52.0	NP_004437.2	67.0
Mus musculus	1e-177	50.7	XP_011237210.1	67.0
Mus pahari	5e-171	50.5	XP_021052971.1	67.0
Bos taurus	2e-179	50.5	NP_001230249.1	71.0
Canis lupus familiaris	2e-174	51.4	XP_849468.1	67.0
Carlito syrichta	1e-175	51.8	XP_008053501.1	67.0
Microcebus murinus	4e-175	51.1	XP_012646889.1	67.0
Papio anubis	7e-174	52.0	XP_021782780.1	67.0

Table S2: Percentage variances of the first five principal components for the holo protein and the ligand-bound complexes.

Protein complex	PC1 (%)	PC2 (%)	PC3 (%)	PC4 (%)	PC5 (%)
PfProRS-ADN	46.54	25.10	13.19	9.17	6.00
PfProRS-SANC184	47.79	20.46	15.37	9.45	6.94
PfProRS-SANC257	63.31	18.12	9.65	4.63	4.30
PfProRS-SANC264	51.50	17.96	12.99	10.59	6.95
PfProRS-SANC456	45.63	22.22	15.52	8.82	7.80
PfProRS-SANC622	46.20	21.36	14.52	10.14	7.77
PfProRS-SANC152	50.44	19.66	13.53	9.02	7.35
PfProRS-SANC235	49.61	20.04	13.91	8.36	8.08
PfProRS-SANC236	46.14	27.14	13.92	7.30	5.50
PfProRS-SANC244	40.48	26.11	16.41	9.16	7.84
PfProRS-SANC318	48.74	21.44	13.68	9.53	6.62

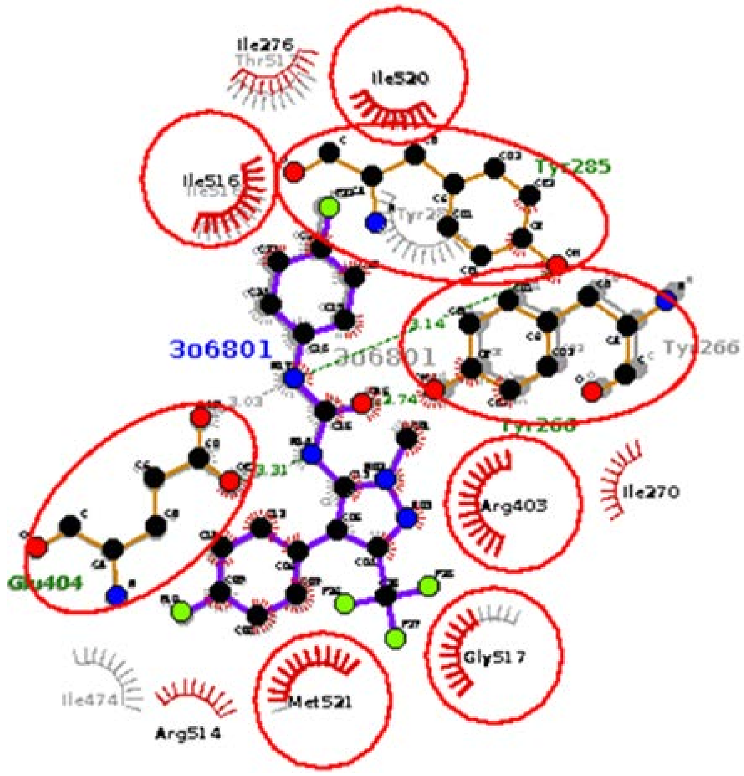

Figure S1: Analysis of ligand-receptor interactions of PfProRS co-crystallized TCMDC-124506 (grey) and the redocked pose (blue). Identical interactions for the co-crystallized ligand and the best docked pose are shown by red ellipses.

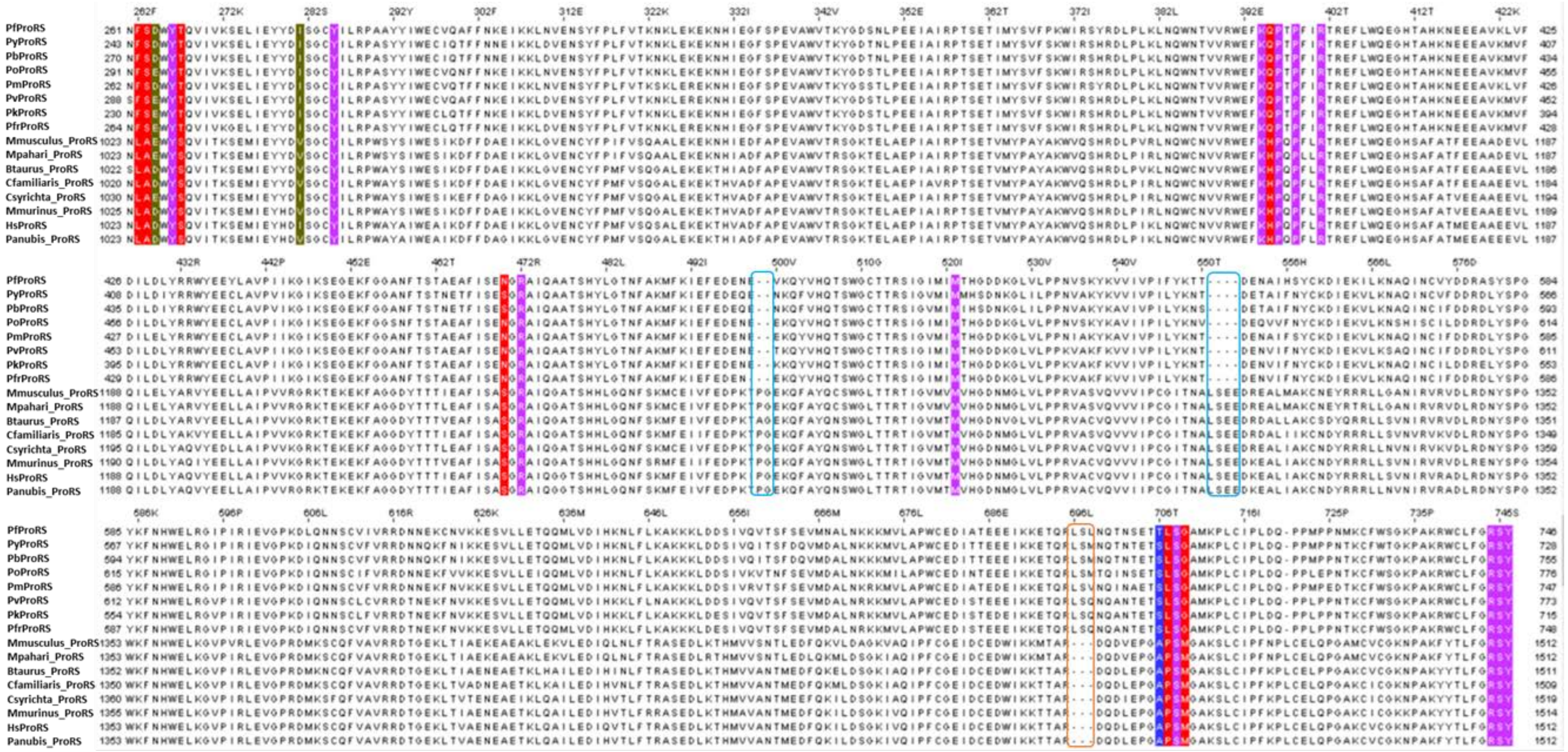

Figure S2: Multiple sequence alignment of selected *Plasmodium* and mammalian ProRS catalytic and anticodon binding domains. Residue numbering is given for *Plasmodium falciparum.* Insertions in *Plasmodium* sequences and mammalian sequences are shown in orange and cyan boxes respectively. Residues implicated in binding of ligands at the allosteric site are shown. Residues conserved in all analysed sequences are shown in magenta, residues conserved in *Plasmodium* but different in mammals are shown in red while residues that were partially conserved are shown in forest green. Residue Thr706 in *P. falciparum* corresponds to a serine in all other *Plasmodium* sequences while for mammalian sequences the corresponding residue is alanine as shown with blue color.

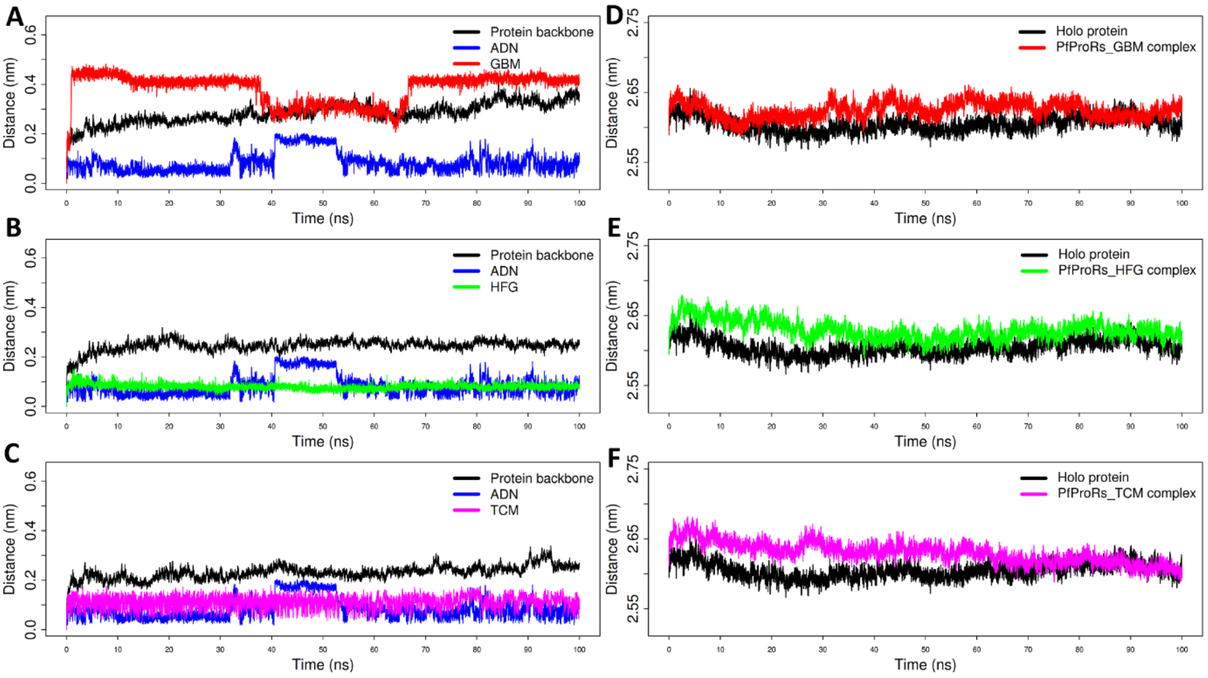

Figure S3. PfProRS backbone RMSD and radius of gyration (Rg) analysis of all atom MD trajectories for known orthosteric and allosteric inhibitors. A) RMSD for PfProRS-Glyburide complex, B) RMSD for PfProRS-halofuginone complex, C) RMSD for PfProRS-TCMDC124506 complex, D) Rg for PfProRS-glyburide complex, E) Rg for PfProRS-halofuginone complex, F) Rg for PfProRS-TCMDC124506 complex.

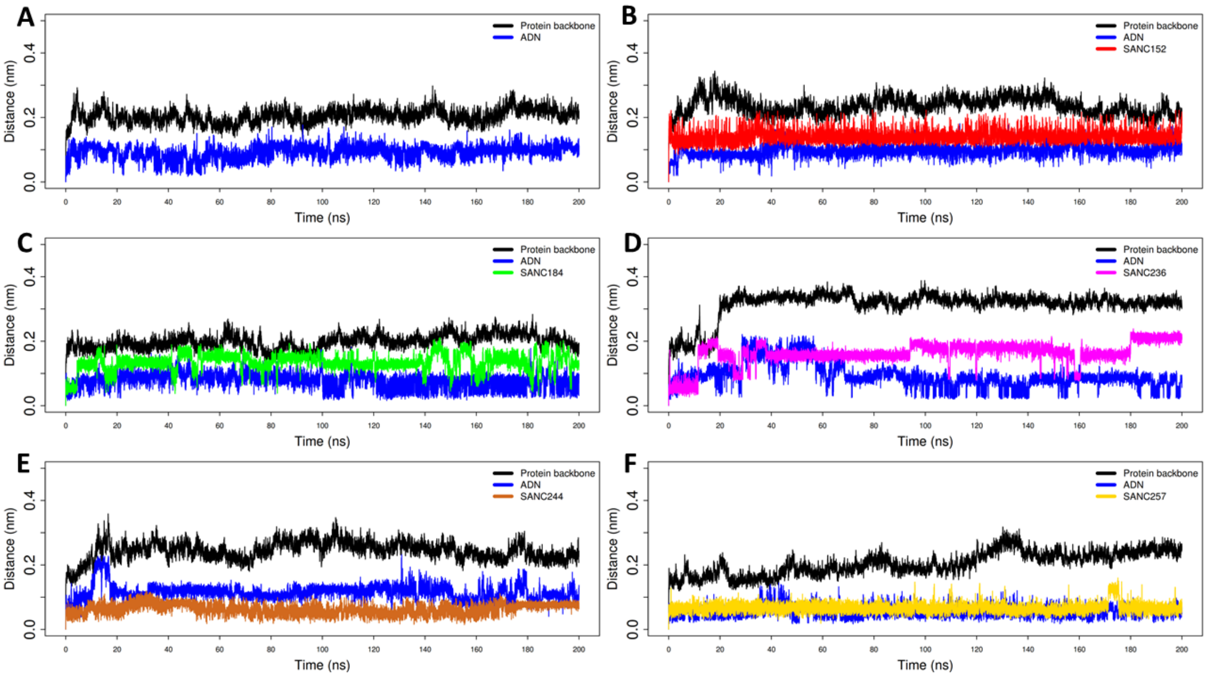

Figure S4: HsProRS backbone RMSD analysis of all atom MD trajectories for the holo system and HsProRS-ligand complexes during the 200 ns simulation. A) Holo system (HsProRS-ADN), B) HsProRS-SANC152 complex, C) HsProRS-SANC184 complex, D)

HsProRS-SANC236 complex, E) HsProRS-SANC244 complex and F) HsProRS-SANC257 complex.

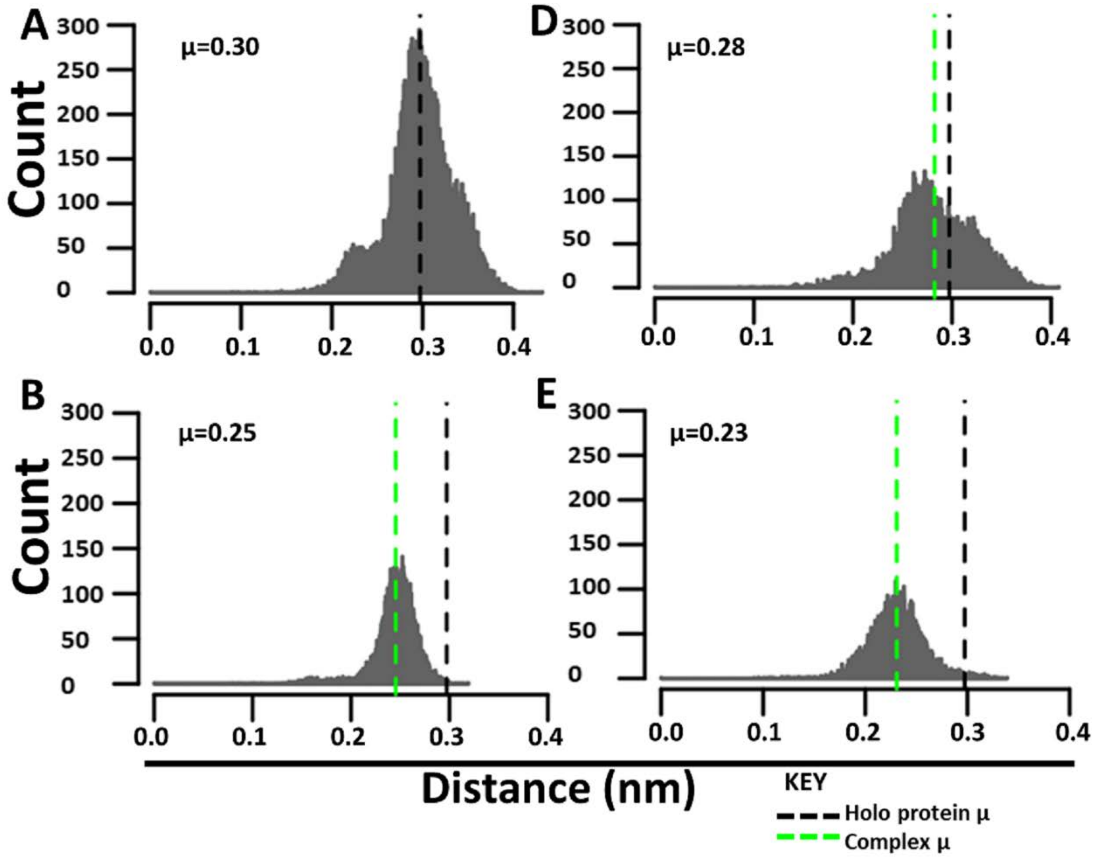

Figure S5: Backbone RMSD distribution plots for PfProRS inhibitor-bound complexes. Conformational flexibility can be assessed by comparing the backbone RMSD distribution of each ligand-bound complex and the holo protein. Comparison of the mean (μ) of the holo protein (black dashed line) to each inhibitor complex (green dashed line) demonstrates the shift in conformation distribution of the complexes during the 100 ns MD simulation. A) Holo system (PfProRS-ADN), B) PfProRS-glyburide complex, C) PfProRS-halofuginone complex and D) PfProRS-TCMDC124506 complex.

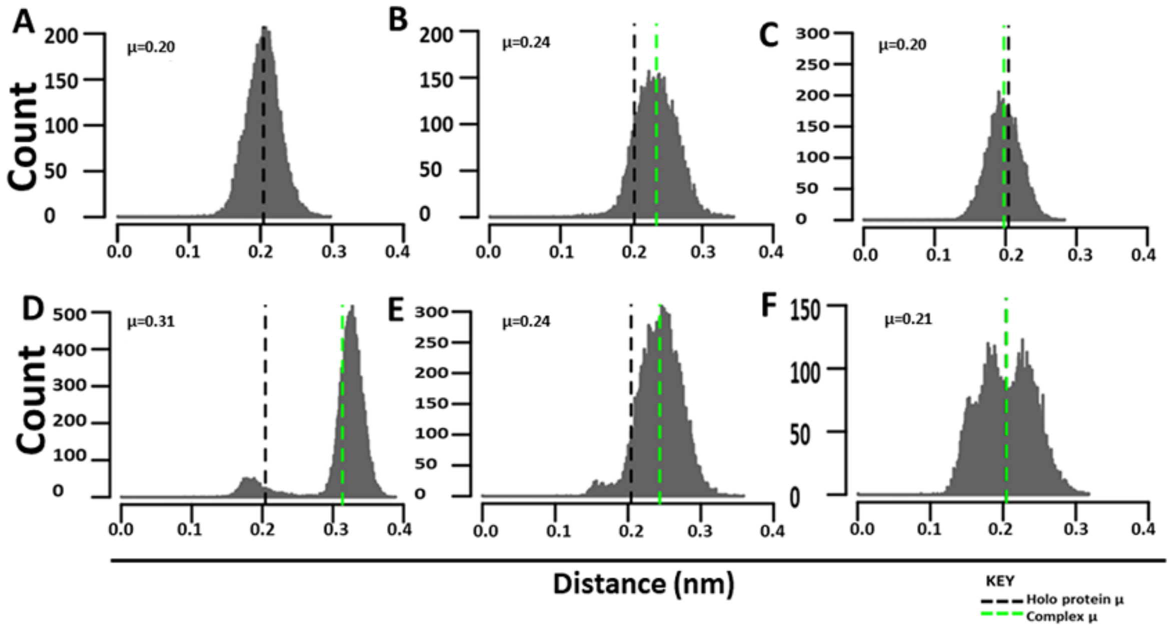

Figure S6: Backbone RMSD distribution plots for HsProRS ligand-bound complexes. Conformational flexibility can be assessed by comparing the backbone RMSD distribution of each ligand-bound complex and the holo protein. Comparison of the mean (μ) of the holo protein (black dashed line) to each ligand complex (green dashed line) demonstrates the shift in conformation distribution of the complexes during the 200 ns MD simulation. A) Holo system (HsProRS-ADN), B) HsProRS-SANC152 complex, C) HsProRS-SANC184 complex, D) HsProRS-SANC236 complex, E) HsProRS-SANC244 complex and F) HsProRS-SANC257 complex.

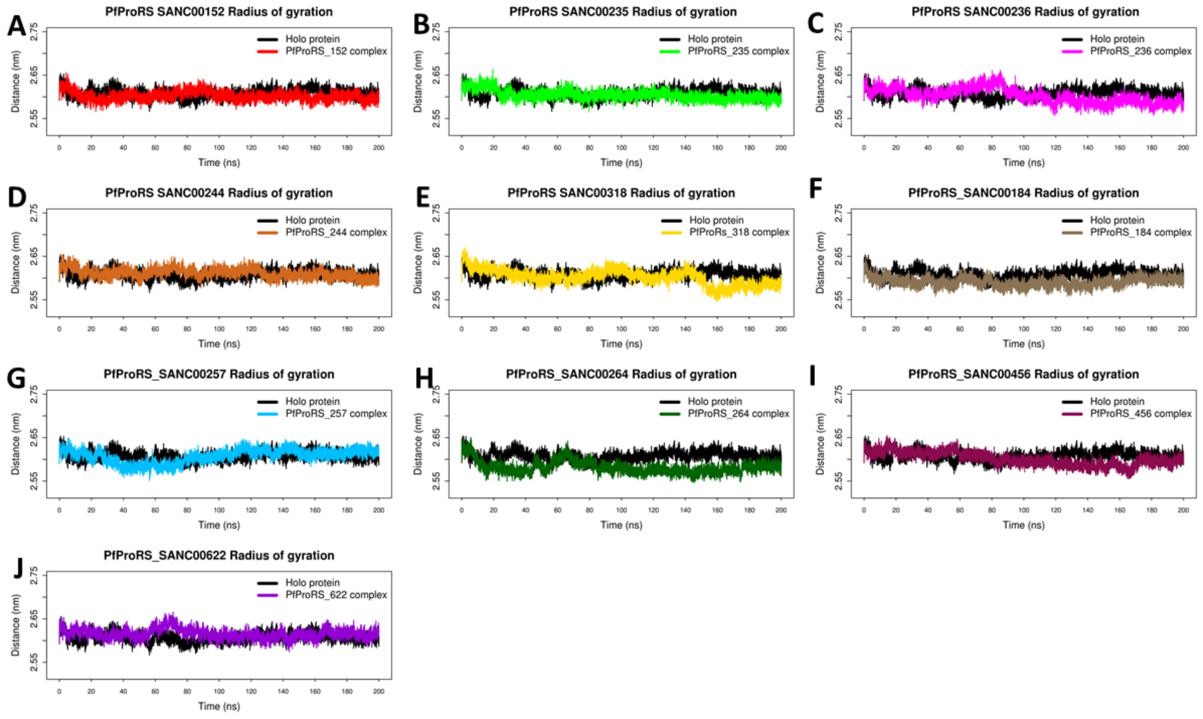

Figure S7: Radius of gyration for the protein backbone of the PfProRS holo protein and ligand-bound complexes over the 200 ns simulation. A) PfProRS-SANC152 complex, B) PfProRS-SANC235 complex, C) PfProRS-SANC236 complex, D) PfProRS-SANC244

complex, E) PfProRS-SANC318 complex, F) PfProRS-SANC184 complex, G) PfProRS-SANC257 complex, H) PfProRS-SANC264 complex, I) PfProRS-SANC456 complex and J) PfProRS-SANC622 complex.

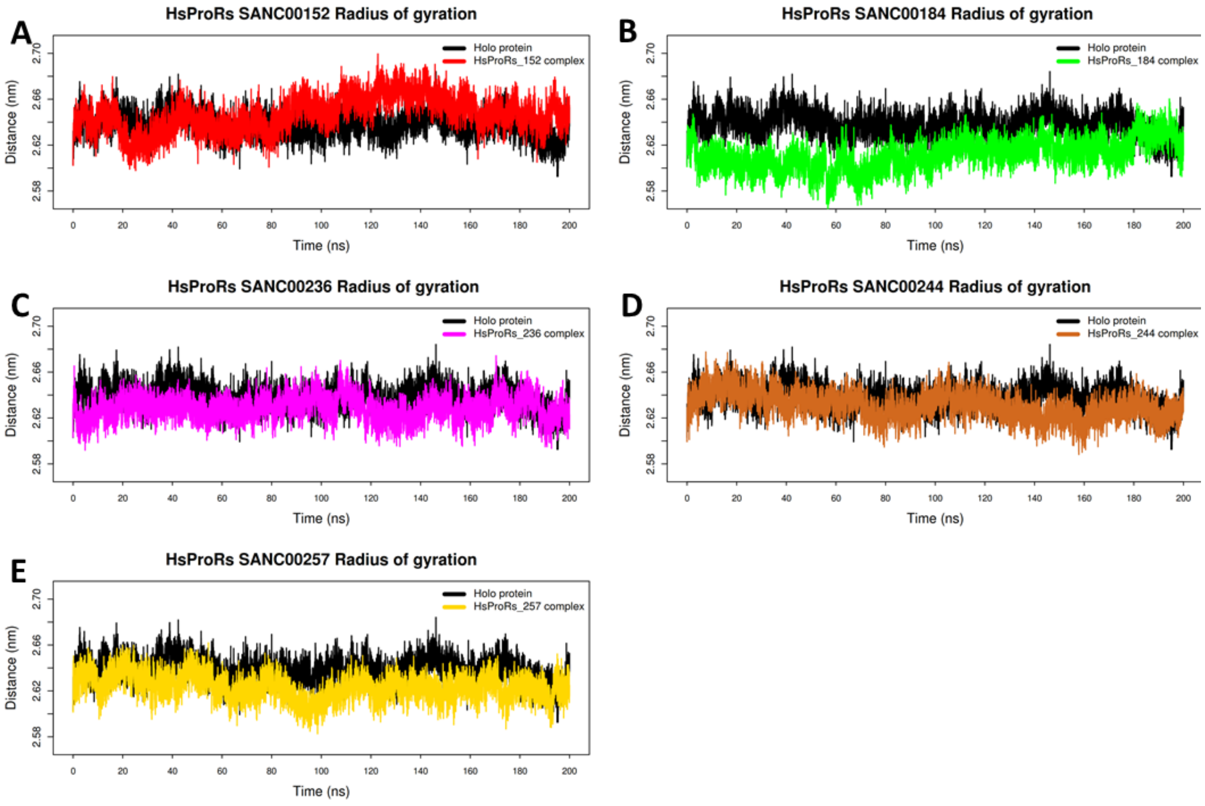

Figure S8: Radius of gyration for the protein backbone of the HsProRS holo protein and ligand-bound complexes over the 200 ns simulation. A) HsProRS-SANC152 complex, B) HsProRS-SANC184 complex, C) HsProRS-SANC236 complex, D) HsProRS-SANC244 complex and E) HsProRS-SANC257 complex.

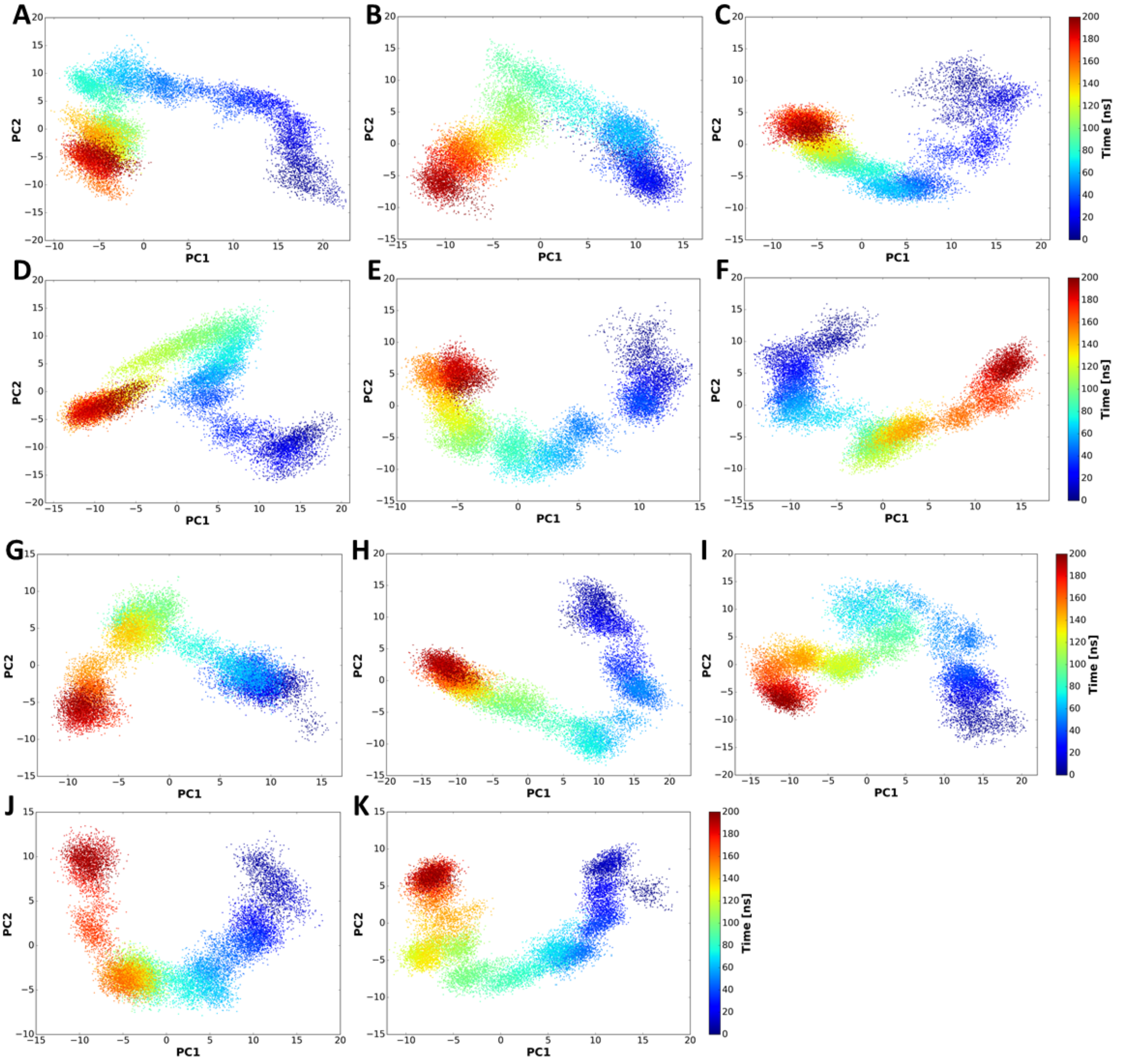

Figure S9. Cartesian coordinate principal component analysis of PfProRS holo protein and PfProRS-ligand bound complexes. The protein motion of all atom MD simulation is shown along the first and second principal components (PC1 and PC2). A) Holo protein (PfProRS-ADN), B) PfProRS-SANC152 complex, C) PfProRS-SANC235 complex, D) PfProRS-SANC236 complex, E) PfProRS-SANC244 complex, F) PfProRS-SANC318 complex, G) PfProRS-SANC184 complex, H) PfProRS-SANC257 complex, I) PfProRS-SANC264 complex, J) PfProRS-SANC456 complex and K) PfProRS-SANC622 complex.

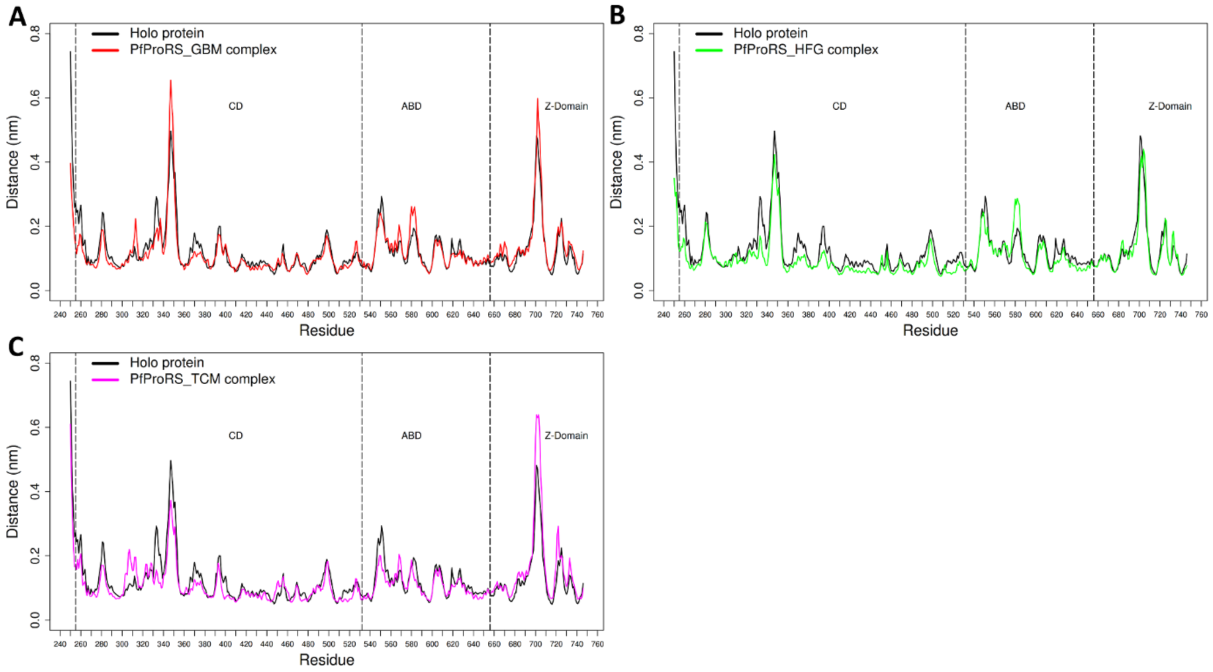

Figure S10: Per residue root mean square fluctuation analysis of the holo system and PfProRS inhibitor-complexes during the simulation. A) PfProRS-glyburide complex, B) PfProRS-halofuginone complex, C) HsProRS-TCMDC124506 complex.

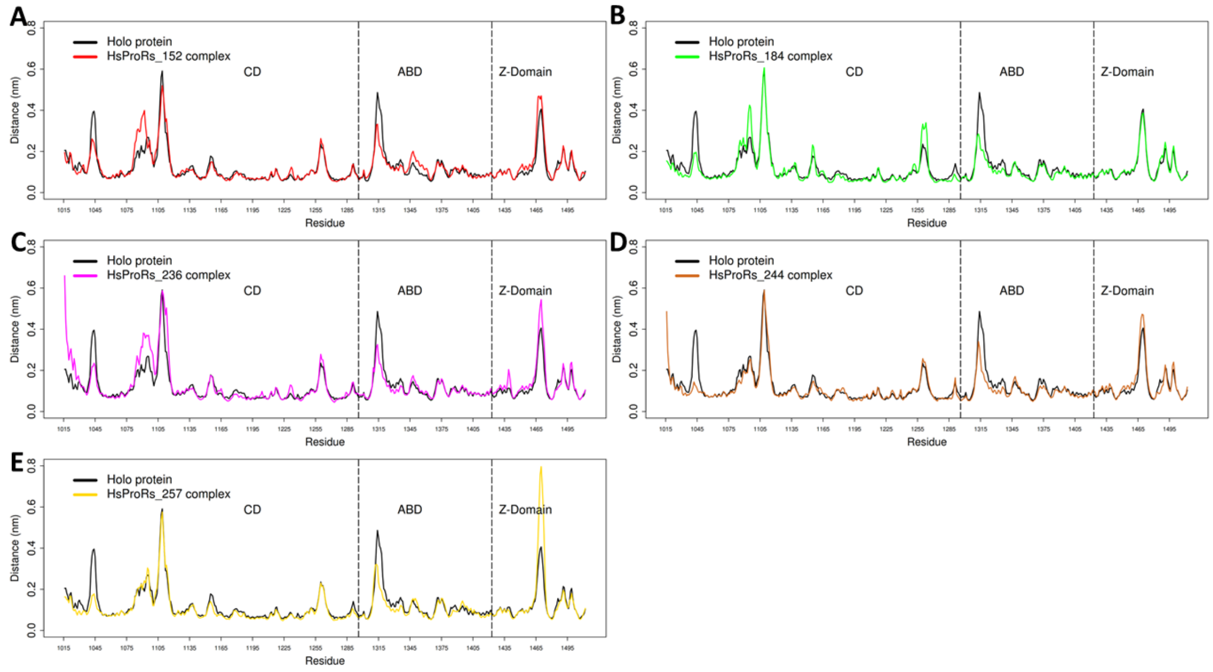

Figure S11: Per residue root mean square fluctuation analysis of the holo system and HsProRS ligand-complexes during the 200 ns simulation. A) HsProRS-SANC152 complex, B) HsProRS-SANC184 complex, C) HsProRS-SANC236 complex, D) HsProRS-SANC244 complex and E) HsProRS-SANC257 complex.

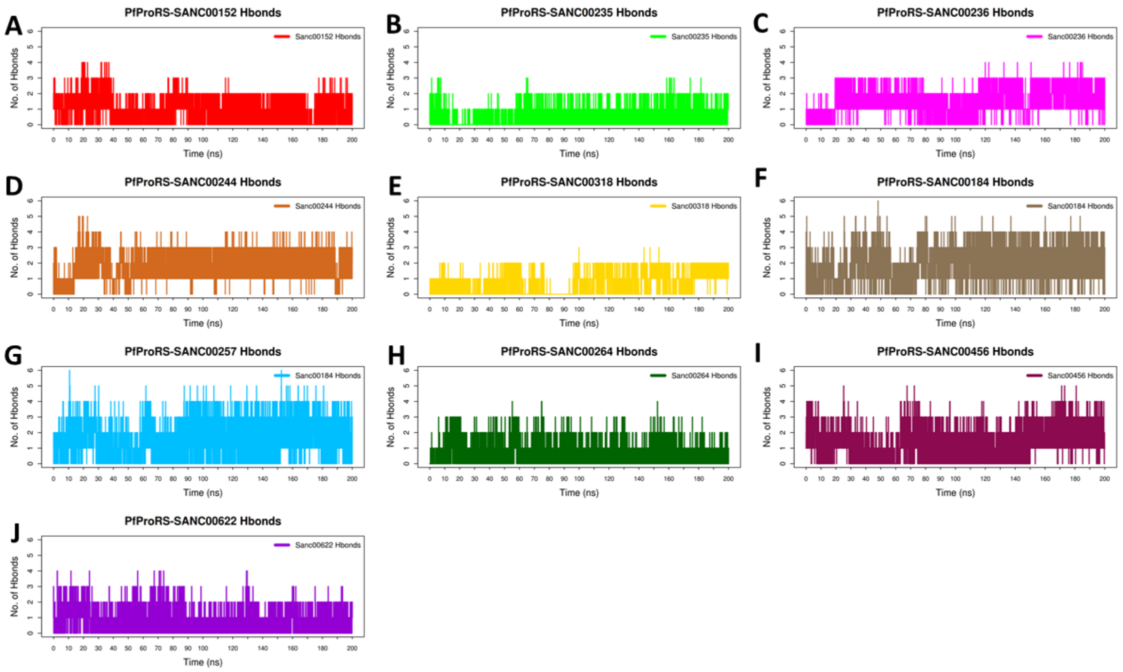

Figure S12: Evolution of hydrogen bond interactions for the PfProRS ligand bound complexes over the 200 ns simulation. A) PfProRS-SANC152 complex, B) of PfProRS-SANC235 complex, C) PfProRS-SANC236 complex, D) PfProRS-SANC244 complex, E) PfProRS-SANC318 complex, F) PfProRS-SANC184 complex, G) PfProRS-SANC257, H) PfProRS-SANC264, I) PfProRS-SANC456 and J) PfProRS-SANC622.

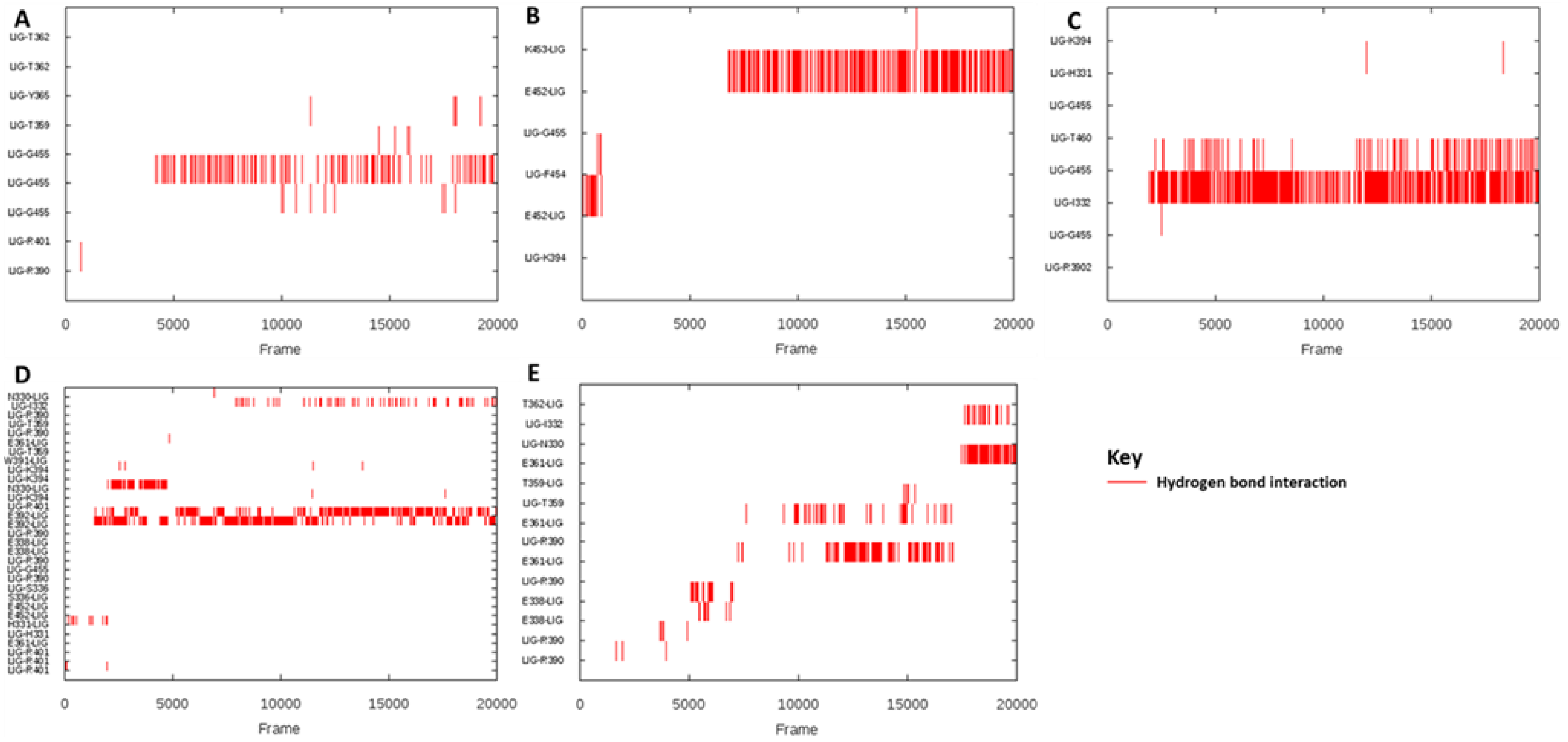

Figure S13: Hydrogen bond interaction between residues at the active site and the selected orthosteric hits. Individual residues interacting with the ligands during the 200 ns MD simulations are shown. The presence of a hydrogen bond interaction is shown by a red line while white spaces show absence of hydrogen bonds. A) Residues interacting with SANC152 in PfProRS-SANC152 complex; B) Residues interacting with SANC235 in PfProRS-SANC235 complex; C) Residues interacting with SANC236 in PfProRS-SANC236 complex; D) Residues interacting with SANC244 in PfProRS-SANC244 complex and E) Residues interacting with SANC318 in PfProRS-SANC318 complex.

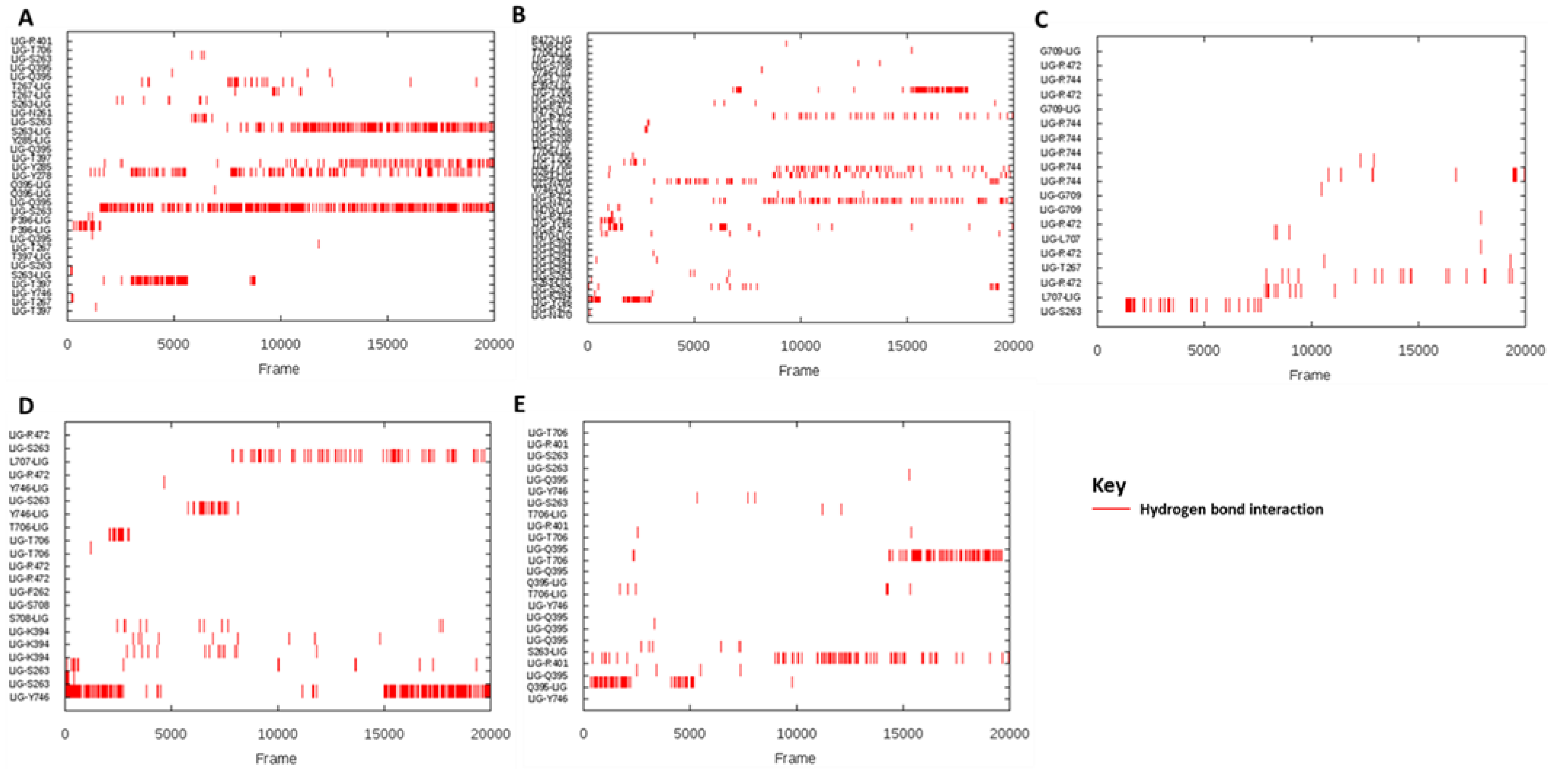

Figure S14: Hydrogen bond interaction between residues at the allosteric site and selected hits. Individual residues interacting with the ligands during the 200 ns MD simulations are shown. The presence of a hydrogen bond interaction is shown by a red line while white spaces show absence of hydrogen bonds. A) Residues interacting with SANC184 in PfProRS-SANC184 complex; B) Residues interacting with SANC257 in PfProRS-SANC257 complex; C) Residues interacting with SANC264 in PfProRS-SANC264 complex; D) Residues interacting with SANC456 in PfProRS-SANC456 complex and E) Residues interacting with SANC622 in PfProRS-SANC622 complex.

Table S3: Pearson's correlation coefficients showing the relationship between *L* vs RMSF, *BC* vs RMSF, *BC* vs inverse *L* and *BC* vs inverse RMSF.

**Ligand**	***L* vs RMSF**	***BC* vs RMSF**	***BC* vs L^{-1}**	***BC* vs $RMSF^{-1}$**
PfProRS-SANC152	0.71	-0.45	0.71	0.60
PfProRS-SANC235	0.63	-0.43	0.71	0.56
PfProRS-SANC236	0.56	-0.39	0.72	0.54
PfProRS-SANC244	0.58	-0.42	0.73	0.57
PfProRS-SANC318	0.59	-0.38	0.72	0.55
PfProRS-SANC184	0.65	-0.40	0.71	0.57
PfProRS-SANC257	0.64	-0.45	0.73	0.53
PfProRS-SANC264	0.54	-0.36	0.73	0.55
PfProRS-SANC456	0.73	-0.48	0.72	0.60
PfProRS-SANC622	0.64	-0.38	0.71	0.55

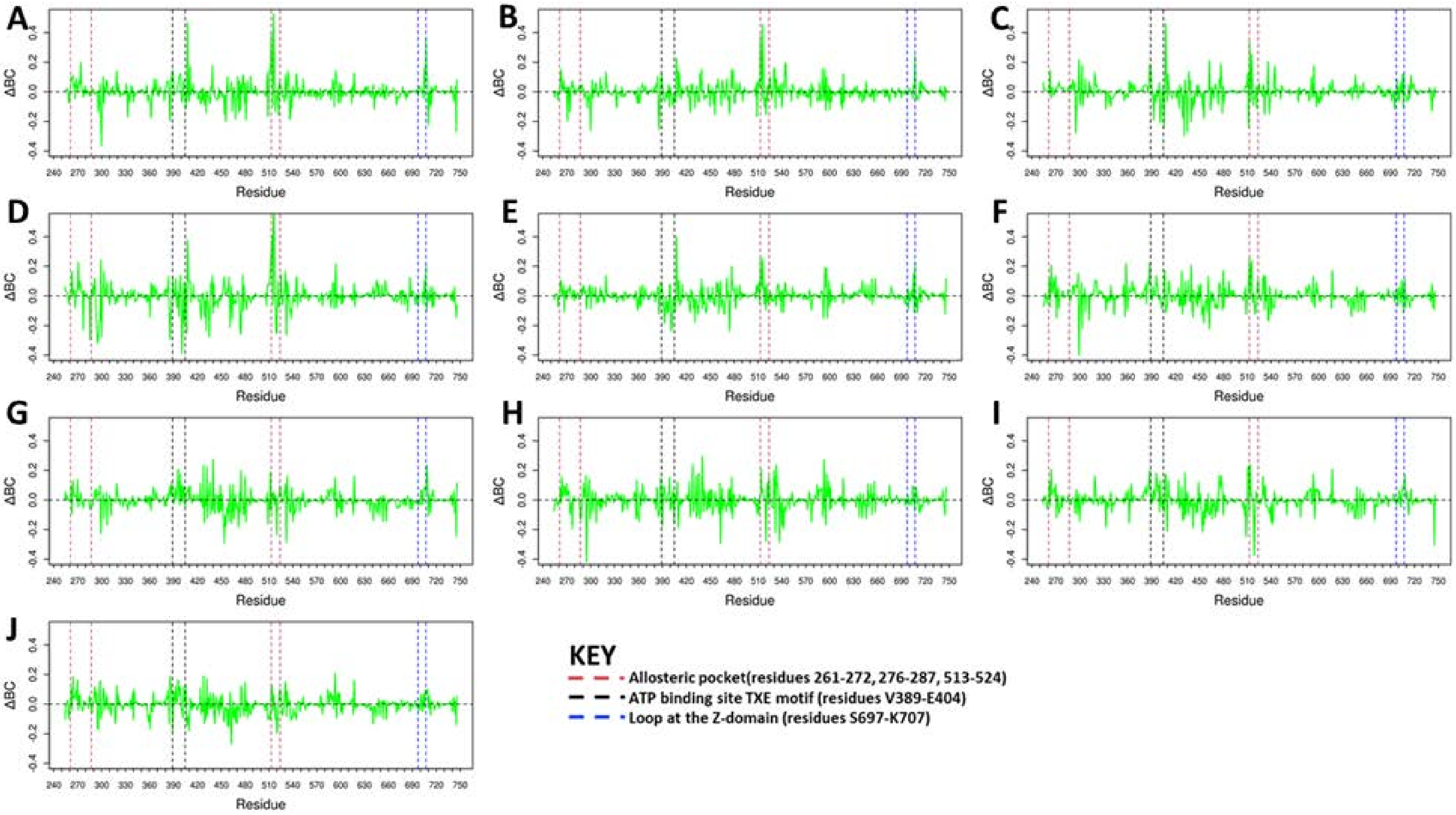

Figure S15: Differences in *betweenness centrality* (*BC*) between the PfProRS holo protein and ligand bound complexes (Holo protein less ligand-bound complex). Residues forming the allosteric pocket are shown in maroon dashed lines, the ATP binding TXE loop is shown in black dashed lines and a loop in the Z-domain is shown in blue dashed lines. A) PfProRS-SANC152 complex, B) PfProRS-SANC235 complex,

C) PfProRS-SANC236 complex, D) PfProRS-SANC244 complex, E) PfProRS-SANC318 complex, F) PfProRS-SANC184 complex, G) PfProRS-SANC257 complex, H) PfProRS-SANC264 complex, I) PfProRS-SANC456 complex and J) PfProRS-SANC622 complex.

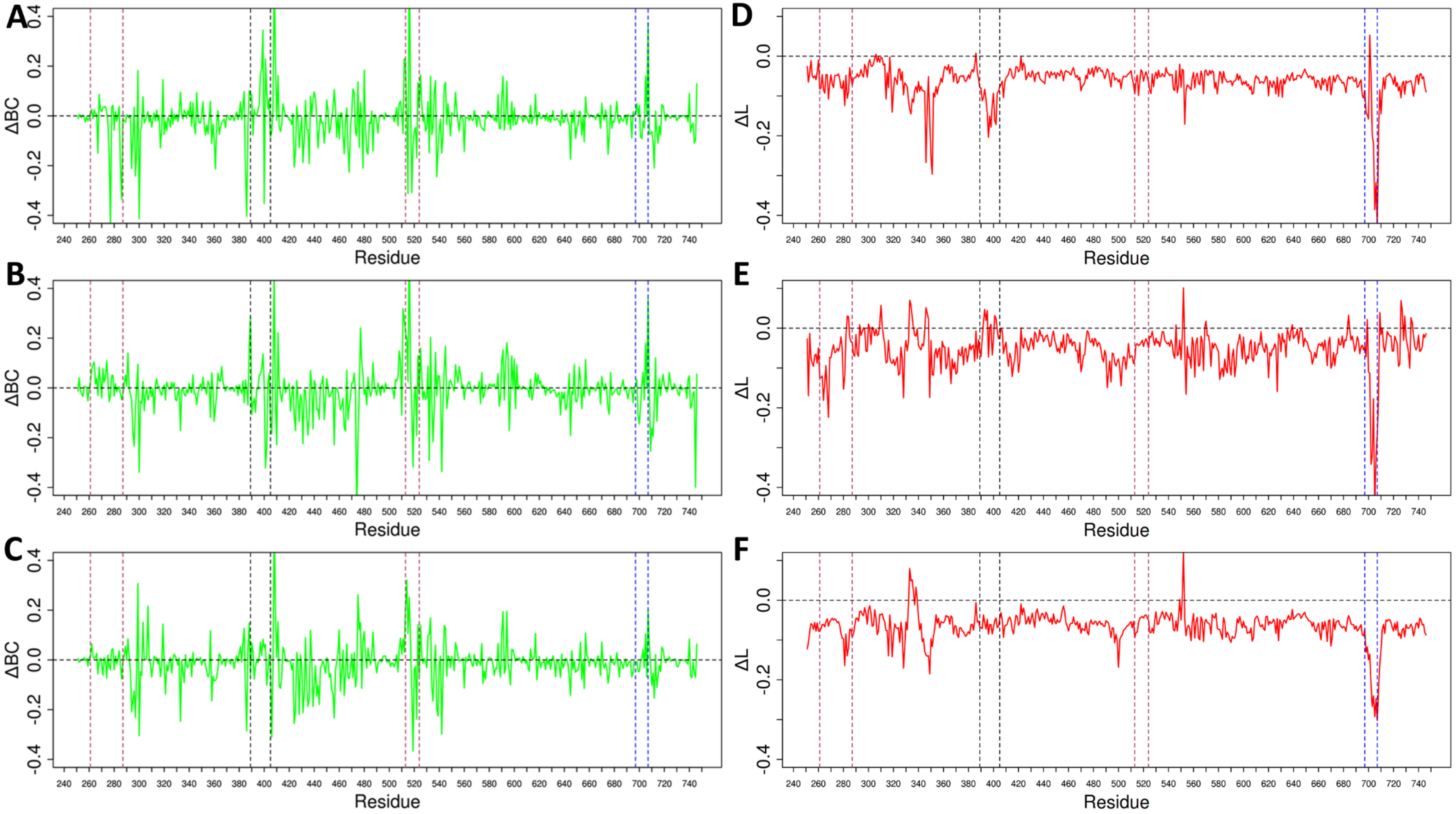

Figure S16: Differences in *betweenness centrality* (*BC*) and average shortest path (*L*) between PfProRS holo protein and inhibitor bound complexes (Holo protein less inhibitor-bound complex). Residues forming the allosteric pocket are shown in maroon dashed lines, the ATP binding TXE loop is shown in black dashed lines and a loop in the Z-domain is shown in blue dashed lines. A) PfProRS-glyburide complex Δ*BC*, B) PfProRS-halofuginone complex Δ*BC*, C) PfProRS-TCMDC124506 complex Δ*BC*, D) PfProRS-glyburide complex Δ*L*, E) PfProRS-halofuginone complex Δ*L* and F) PfProRS-TCMDC124506 complex Δ*L*.

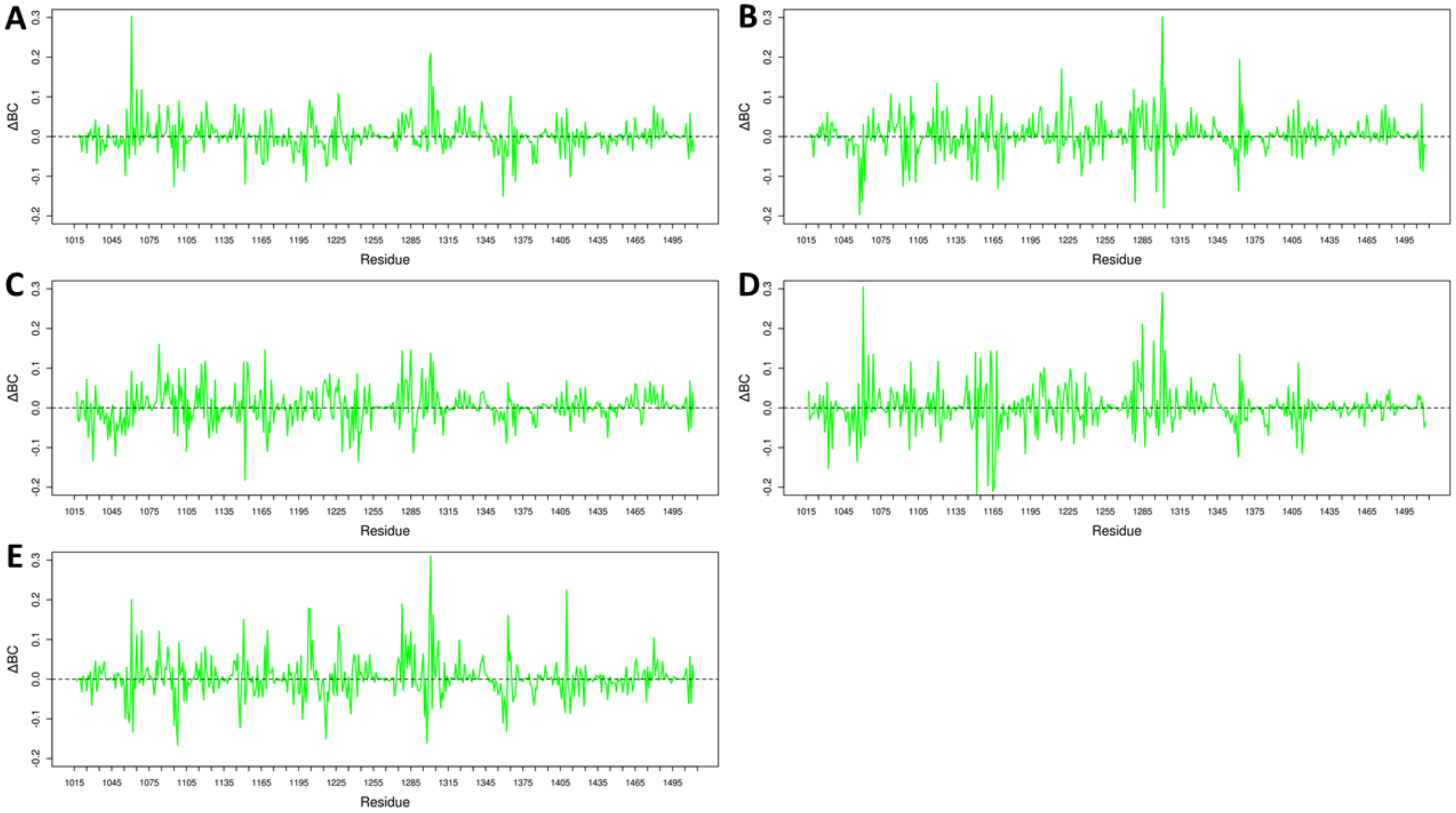

Figure S17: Differences in *betweenness centrality* (*BC*) between the HsProRS holo protein and ligand bound complexes (Holo protein less ligand-bound complex). A) HsProRS-SANC152 complex, B) HsProRS-SANC184 complex, C) HsProRS-SANC236 complex, D) PfProRS-SANC244 complex and E) PfProRS-SANC257 complex.

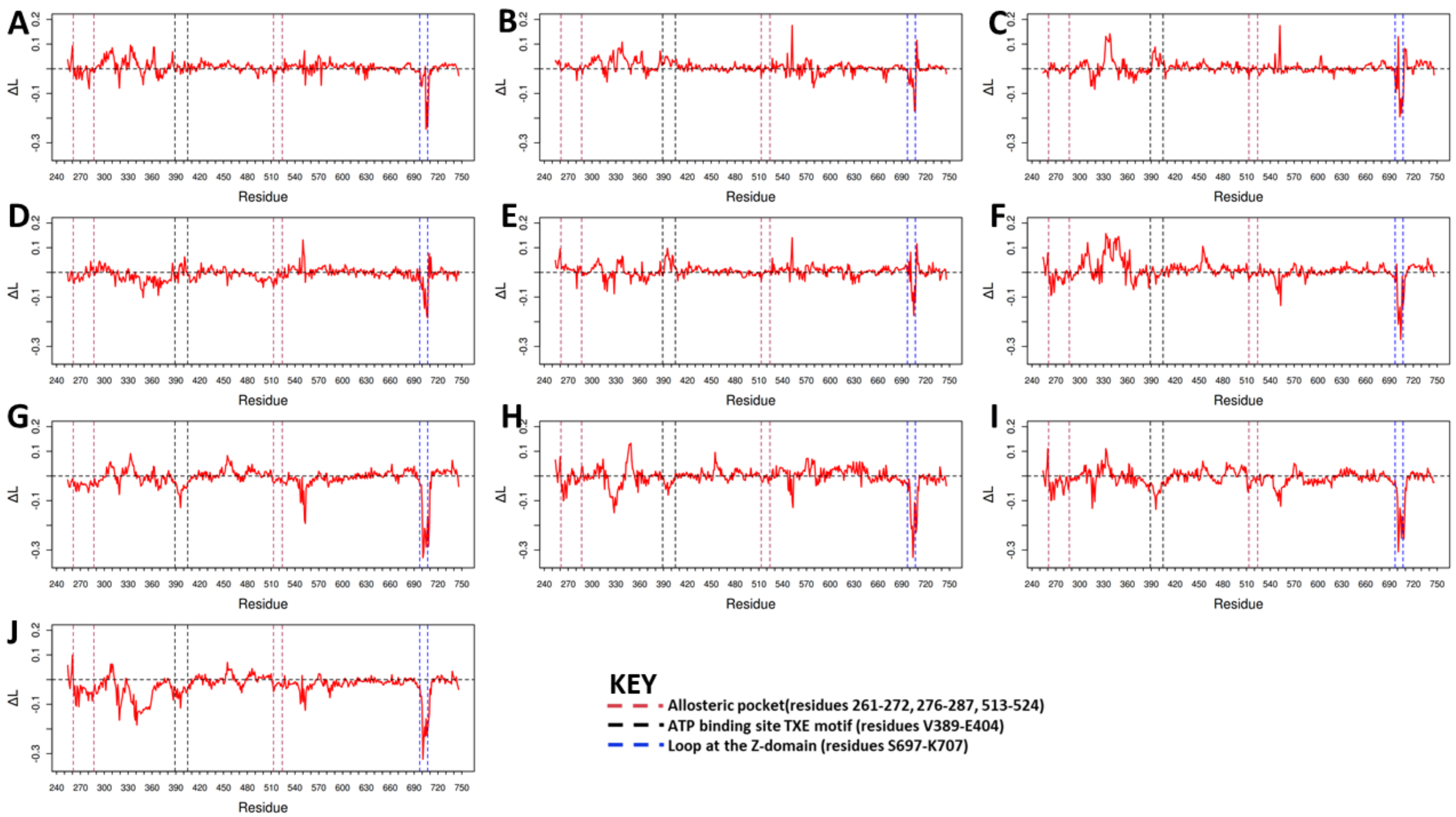

Figure S18: Differences in average shortest path (*L*) between the holo protein and PfProRS ligand bound complexes (Holo protein less ligand-bound complex). Residues forming the allosteric pocket are shown in maroon dashed lines, the ATP binding TXE loop is shown in black dashed lines and a loop in the Z-domain is shown in blue dashed lines. A) PfProRS-SANC152 complex, B) PfProRS-SANC235 complex, C) PfProRS-SANC236 complex, D) PfProRS-SANC244 complex, E) PfProRS-SANC318 complex, F) PfProRS-SANC184 complex, G) PfProRS-SANC257 complex, H) PfProRS-SANC264 complex, I) PfProRS-SANC456 complex and J) PfProRS-SANC622 complex.

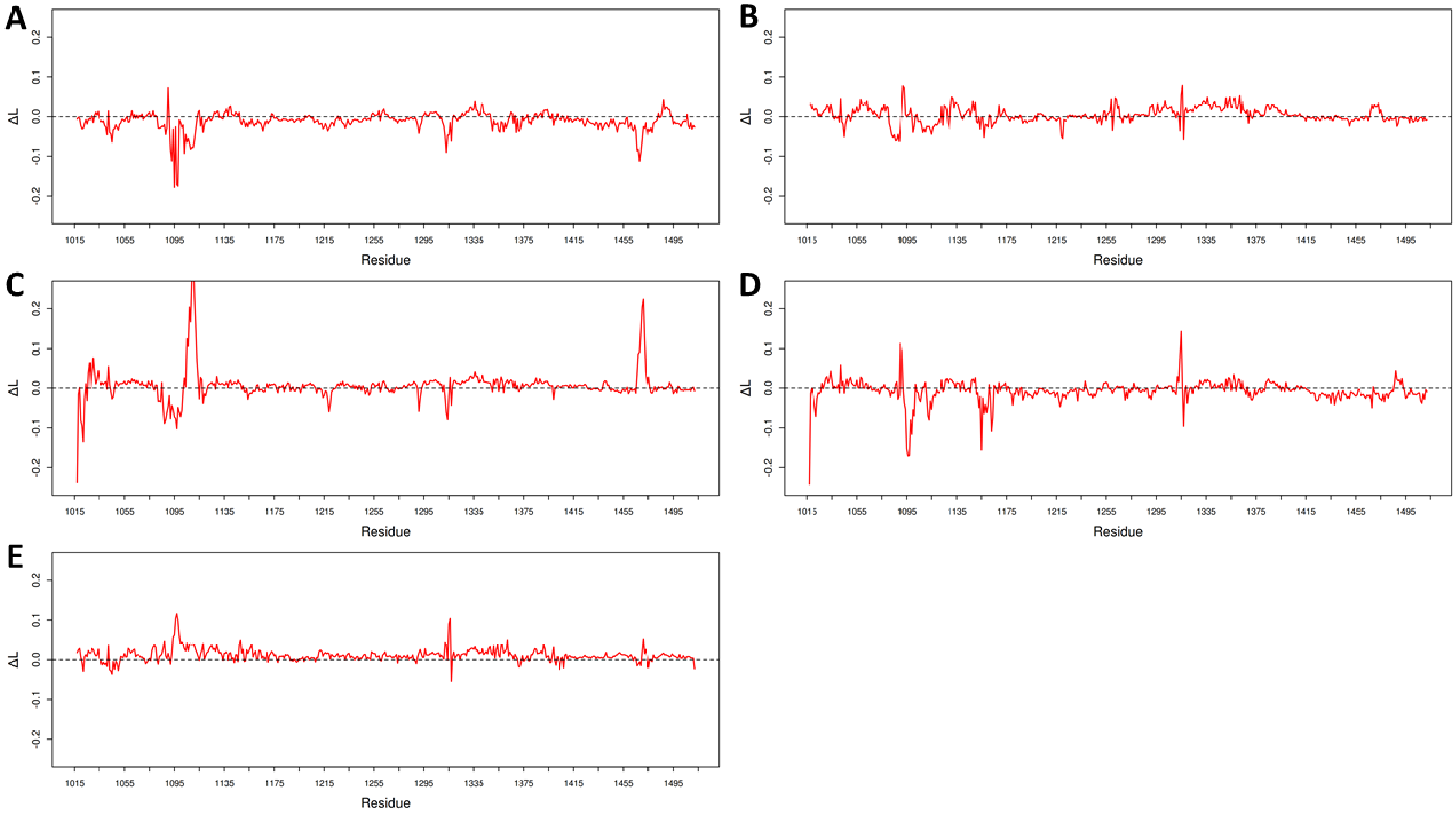

Figure S19: Differences in average shortest path (*L*) between HsProRS holo protein and ligand bound complexes (Holo protein less ligand-bound complex). A) HsProRS-SANC152 complex, B) HsProRS-SANC184 complex, C) HsProRS-SANC236 complex, D) HsProRS-SANC244 complex and E) HsProRS-SANC257 complex.
